# A comprehensive overview of the biological foundations of ADHD

**DOI:** 10.1042/BSR20254061

**Published:** 2026-06-10

**Authors:** Isabella Folego-Temoteo, Yago C. Lima, Eugenio H. Grevet, Marcos V. Vidor, Maria Eduarda de A. Tavares, Bruna S. da Silva, Claiton H.D. Bau, Diego L. Rovaris

**Affiliations:** 1 Laboratory of Physiological Genomics of Mental Health (PhysioGen Lab), Instituto de Ciencias Biomedicas, Universidade de Sao Paulo, São Paulo, Brazil; 2 ADHD Outpatient Program & Developmental Psychiatry Program, Hospital de Clínicas de Porto Alegre, Federal University of Rio Grande do Sul, Porto Alegre, Brazil; 3 Universidade Federal do Rio Grande do Sul, Faculdade de Medicina, Departamento de Psiquiatria e Medicina Legal, Programa de Pós-Graduação em Psiquiatria e Ciências do Comportamento, Porto Alegre, RS, Brazil; 4 Universidade Federal de Ciências da Saúde, Departamento de Genética, Porto Alegre, RS, Brazil; 5 Universidade Federal do Rio Grande do Sul, Instituto de Biociências, Departamento de Genética, Programa de Pós-Graduação em Genética e Biologia Molecular, Porto Alegre, RS, Brazil

**Keywords:** Attention-Deficit/Hyperactivity Disorder, Gene–environment interaction, Genomics, GWAS, Neurodevelopmental disorders, Neuroimaging, Neurotransmitters, Omics approaches

## Abstract

Attention-deficit/hyperactivity disorder (ADHD) is a neurodevelopmental condition marked by persistent and impairing patterns of inattention, hyperactivity, and impulsivity. Evidence from neurochemical, pharmacological, and genetic research supports the hypothesis that ADHD involves alterations in neurotransmission, primarily within dopaminergic and noradrenergic systems, with contributions from other neurotransmitter pathways and their interactions. Neuroimaging studies identify structural and functional differences in regions such as the frontal cortex and subcortical structures, although findings remain heterogeneous. Genomic research indicates a polygenic basis, with common and rare variants influencing synaptic transmission, neuronal development, regulatory pathways, and related biological processes. These studies also point to shared genetic influences between ADHD and psychological, social, and somatic traits. Additional omics approaches have further expanded these insights, although larger and more integrative studies across multiple layers remain needed. Environmental factors not only influence the onset of ADHD but also shape its course and prognosis, with emerging evidence highlighting complex gene-environment correlations and interactions. Together, the heterogeneity of findings across neuroimaging, genomic, and multi-omics studies underscores the importance of integrative approaches that embrace diversity across populations, methodologies, and biological systems. The present review provides a comprehensive overview of ADHD's biological foundations, highlighting central nervous system mechanisms, their interplay with genetic and environmental factors, and recent advances from multi-omics research with translational potential. We also discuss key methodological considerations, emphasizing that the biological architecture of ADHD is complex, highly polygenic, and spans multiple levels of analysis.

## Introduction

**Figure 1:**
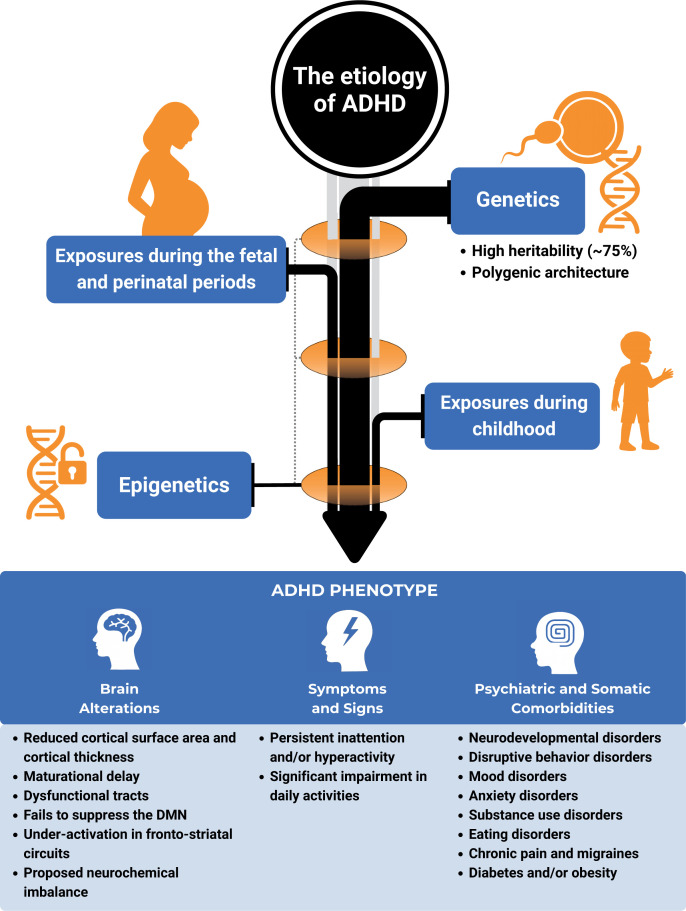
Integrative biological framework of ADHD across development ADHD emerges from the interaction between polygenic liability and environmental exposures acting early in brain development. The genetic contribution is among the highest observed in neuropsychiatric disorders. These influences converge on multiple neurobiological systems, including hypothesized neurotransmitter signaling alterations, brain network organization (e.g., the default mode network, DMN), and neurodevelopmental trajectories, whose effects unfold dynamically across the lifespan. Rather than reflecting a single causal pathway, the model highlights how heterogeneous genetic and environmental factors shape shared downstream biological processes, contributing to variability in symptom expression, persistence, and vulnerability to ADHD-related outcomes. The figure represents a conceptual framework rather than a definitive mechanistic model.

Attention-deficit/hyperactivity disorder (ADHD) is a common neurodevelopmental condition characterized by persistent and clinically impairing inattention and/or hyperactivity-impulsivity [1–3] (Figure 1). It affects executive functioning, frequently persists across the lifespan, and is associated with educational, occupational, and social impairments, as well as increased psychiatric and somatic comorbidities [3–12].

Prevalence is estimated at 7.2% in children [13] and 2.6% in adults [14] but rises sharply in clinical settings, where pooled estimates reach ∼32% in pediatric and ∼21% in adult populations [15]. Despite this, substantial diagnostic and treatment gaps persist worldwide, particularly in low- and middle-income countries [16]. Strong evidence supports its neurobiological basis, and scientific consensus has established its clinical validity [17], highlighting ADHD as a major public health challenge that demands ongoing research to elucidate its underlying mechanisms, improve clinical management, and implement more equitable strategies for diagnosis and treatment across all populations.

In the present review, we provide a narrative and integrative synthesis of the biological aspects of ADHD. The literature was selected to represent major advances, consensus findings, and areas of active debate across neurochemistry, neuroimaging, genomics, and related domains, prioritizing meta-analyses, systematic reviews, and large-scale investigations whenever available. When inconsistencies emerged, they were interpreted in light of methodological heterogeneity, sample characteristics, developmental stage, and study design. Smaller or earlier studies were included primarily to provide historical perspective or to illustrate the emergence of mechanistic hypotheses and should be interpreted within that framework.

### ADHD as a neurodevelopmental trait—from behavioral observation to biology and back again

One of the earliest descriptions potentially related to ADHD dates back to ancient Greece [18]. In *Characters*, Theophrastus (372–287 BC) portrays *Anaisthetos* (ἀναίσθητος), often translated as ‘the obtuse man.’ The characteristics used to describe this individual resemble the ADHD symptoms listed in the most recent edition of the Diagnostic and Statistical Manual of Mental Disorders (DSM-5) [2]. In a diagnostic exercise, Theophrastus’ *Anaisthetos* would very likely receive an ADHD diagnosis if he lived today, lending support to the transhistorical stability of the phenotype [18,19]. This observation challenges the common intuition that inattention is primarily a product of modern life—such as screen exposure, fatigue, or contemporary social demands—by indicating that impairing attentional difficulties have long been part of the human behavioral repertoire.

The first medical descriptions emerged in the late 18th century. In 1775, Melchior Adam Weikard referred to *Mangel der Aufmerksamkeit* (‘lack of attention’) to describe individuals with distractibility and difficulty sustaining focus [20]. A few decades later, in 1798, Alexander Crichton offered a more elaborate account of attentional disorders in his treatise *An Inquiry into the Nature and Origin of Mental Derangement*, highlighting the incapacity to maintain attention with constancy. Both works described deficits in attention as a primary disorder, correlating well with the modern concept of ADHD [21].

The modern clinical characterization of the disorder in children began in the early 20th century. In his 1902 Goulstonian Lectures’ ‘Some abnormal psychical conditions in children,’ George Frederic Still systematically described a group of 23 children with normal intellect presenting with aggressive behavior, conduct problems, poor impulse control, learning disorders, and inattention [22]. This account is widely regarded as the first modern clinical characterization of ADHD in children. However, Still’s description was notably broad, outlining an over-inclusive syndrome that grouped symptoms now classified separately under neurodevelopmental disorders and disruptive, impulse-control, and conduct disorders [2].

Still’s comprehensive description gave way to theories that sought a neurological origin, reflecting an early and sustained effort to identify a biological etiology for the condition. The nomenclature evolved following observations of a similar syndrome in child survivors of Von Economo’s encephalitis, which gave rise to the concept of ‘*Brain Damage*.’ Subsequently, the absence of observable neurological damage in many children led to the adoption of a more functional denomination, ‘minimal brain dysfunction’ (MBD) [23], a term that predominated until the late 1980s.

However, the clinical application of MBD proved challenging due to its broad and heterogeneous nature, which limited its diagnostic precision. This ambiguity created a compelling need to disaggregate the overarching concept into more specific clinical entities. The work of researchers like Franz Kramer and Hans Pollnow, by systematically defining a ‘hyperkinetic disorder of childhood,’ exemplified the evidence-based framework required for this subdivision [24,25]. This movement provided a strong rationale for breaking down the MBD concept into its core components, leading to the emergence of the hyperkinetic syndrome and specific learning disorders as two of the first and most clearly defined subsyndromes to be recognized. This process of specification culminated in the disorder’s first formal entry in the DSM-II in 1968 as the ‘Hyperkinetic Reaction of Childhood,’ which emphasized hyperactivity symptoms [26].

As research on the topic expanded [27], the diagnosis was revised. In 1980, DSM-III [28] incorporated inattentive symptoms, and the condition was renamed ‘attention deficit disorder with or without hyperactivity.’ Continued research, now also involving adolescents and adults, led the fourth edition of the DSM to recognize the persistence of symptoms into adulthood and to introduce ADHD subtypes: inattentive, hyperactive-impulsive, or combined [29]. In 2013, with growing clinical and biological research, the DSM-5 revised the age-of-onset criterion (from before age 7 to before age 12), updated the number of symptoms required for diagnosis of the disorder in adults (from 6 to 5), and modified the terminology for clinical presentations, which were previously called subtypes [2]. The core symptoms of ADHD are inattention and/or hyperactivity-impulsivity, which define the predominantly inattentive, predominantly hyperactive/impulsive, and combined presentations.

The current ADHD diagnosis consists of five criteria [30]. The first criterion lists nine inattentive and nine hyperactive/impulsive symptoms; children must show at least six of either set for 6 months. For older adolescents and adults (age 17 and older), at least five symptoms are required. The second criterion asks whether symptoms were present before the age of 12, clearly targeting adults. The third criterion requires that symptoms occur in at least two different settings (e.g., at home and at school/work). The fourth demands clear evidence that symptoms interfere with or reduce the quality of social, academic, or occupational functioning. The fifth excludes the possibility that symptoms are better explained by another condition, such as schizophrenia [2].

Among children and adolescents, ADHD is more common in males than females, with a ratio of 4:1 in those seeking treatment [31]. In adulthood, the sex difference is smaller, with an almost 1:1 ratio [32]. There is no consensus on why these ratios change across the lifespan. However, some hypotheses involve differences in behavior between sexes that facilitate diagnoses in boys, pubertal timing, hormonal changes, and socio-cultural factors [33]. Importantly, recent population-based evidence indicates that females diagnosed later in adolescence or adulthood often already exhibit mental health, educational, and healthcare-related difficulties during childhood, suggesting that delayed diagnosis reflects under-recognition rather than a later-emerging phenotype [34].

The concept of neurodevelopment in ADHD has always been based on the idea that its onset occurs before puberty, and therefore an age-of-onset criterion is included to confirm diagnosis in adults. However, three longitudinal studies have shown that up to 80% of adults with ADHD did not exhibit the disorder during childhood, raising the possibility of a late-onset ADHD subtype [35–37]. A reanalysis of one of those earlier studies [36], using statistical approaches based on clinical criteria and machine learning, found that most adults with the disorder have persistent ADHD—i.e., a neurodevelopmental trajectory—but a subgroup (20%) shows a late-onset course [4]. Adding complexity to this clinical landscape, a DSM-5 field trial showed that ADHD prevalence rates were 2.1% for DSM-5 ADHD full criteria and 5.8% for ADHD disregarding the age of onset criterion [9]. This late-onset subgroup has attracted growing research interest, particularly regarding their potential treatment needs, as they meet the criterion of significant impairment.

**Figure 2:**
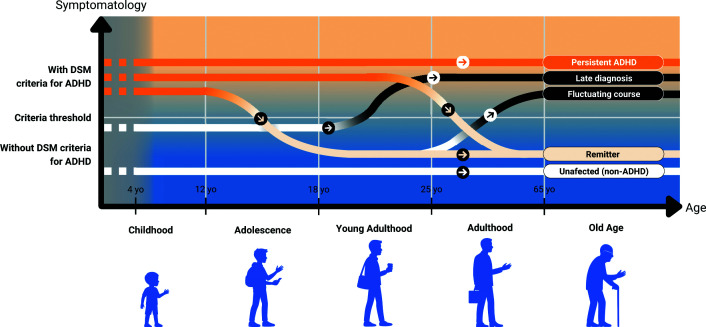
Trajectories of ADHD throughout the lifespan The *y*-axis depicts symptomatology in relation to the Diagnostic and Statistical Manual of Mental Disorders criteria threshold, and the *x*-axis depicts age from childhood to old age. Orange lines represent the first ADHD courses described in the literature: remission across the lifespan (declining below threshold) and persistent ADHD throughout life (remaining above threshold). Early longitudinal cohorts documented age-related decline in ADHD symptomatology from childhood into adulthood [43,44]. In follow-up studies within adulthood, about one-third of the individuals with ADHD achieved diagnostic remission despite comorbidity [6,45]. Factors associated with persistent ADHD include female gender, greater symptom severity, frequent comorbidities, and polygenic liability to depression [5]. Additional courses described in more recent works include late-diagnosed ADHD (symptoms rise to cross the threshold in adolescence/adulthood) and a fluctuating course with periods of remission and recurrence. Late diagnosis has been associated with female gender and higher intelligence quotient (IQ) [4]. At least four longitudinal studies have documented a fluctuating ADHD course [5,38,46,47]. Genetic correlations are high across subgroups: ∼0.8 between childhood and persistent ADHD, ∼0.8 between persistent and late-diagnosed ADHD, and ∼0.7 between childhood and late-diagnosed ADHD [41,42].

The variability in ADHD onset (Figure 2) and course—including the possibility of remission during adolescence [38], persistence [39], adult onset [4,35–37], and adult remission or symptom fluctuation [5–7]—has raised important questions about its pathophysiology. One example is the debate over whether late-onset ADHD represents a neurodevelopmental condition with delayed phenotypic expression, potentially influenced by environmental factors that modulate the emergence of symptoms [40]. This discussion highlights the dynamic interplay between phenotype, environment, and biology. Although late-onset cases challenge the traditional neurodevelopmental framework, evidence from genomic studies suggests that the genetic correlations among childhood, persistent, and late-onset ADHD are largely similar (discussed later) [41,42]. These findings indicate that many individuals with a late-onset diagnosis may represent phenotypic variation within a shared neurodevelopmental trajectory.

Beyond its heterogeneous developmental course, a defining feature of ADHD is its high level of comorbidity with both psychiatric and somatic conditions. In childhood, when hyperactivity tends to be more prominent, frequent comorbidities include oppositional defiant disorder, conduct disorder, anxiety disorders, specific phobias, and enuresis, all of which occur at higher rates than in the general population [48]. Other neurodevelopmental conditions, including autism and dyslexia, are also commonly observed among children and adolescents with ADHD [2]. Clinical presentation changes across the lifespan. Hyperactivity often declines with age, whereas inattention, disorganization, and emotional dysregulation tend to become more prominent in adulthood [49,50]. In parallel, the comorbidity profile shifts, with substance use, mood, anxiety, and personality disorders more frequently reported among adults with ADHD [51]. In addition to psychiatric conditions, several somatic diseases are significantly associated with ADHD, including neurological disorders (e.g., Alzheimer’s and Parkinson’s disease), sleep disturbances, autoimmune diseases, and metabolic outcomes such as obesity, type 2 diabetes, and metabolic syndrome, although the magnitude of these associations varies across conditions [52].

The idea that ADHD is highly comorbid sometimes generates controversy about the diagnosis, with some arguing that it is not truly ADHD but rather another condition. Although genetic studies have shown significant overlap between ADHD and other psychiatric and somatic conditions, these genetic correlations are far from perfect [53–55]. A significant portion of the genetic risk is shared, but there also appears to be a component more strongly related to ADHD, supporting its characterization as a distinguishable neurodevelopmental trait existing along a spectrum of biological and behavioral dimensions.

Overall, the historical evolution of ADHD reflects increasing efforts to define a phenotype that is biologically informative, alongside advances in clinical observation and research methodologies spanning neurochemistry, neuroimaging, genetics, and more recently, omics approaches. Yet, despite these advances, neither ADHD nor psychiatry more broadly has established validated biological markers or endophenotypes that reliably guide diagnosis or treatment, underscoring the challenges of translating biological discoveries into therapeutic decision-making. In line with this research trajectory, the following sections are organized to first examine neurochemistry, followed by neuroimaging, genomics, and other omics approaches, and finally their translation into clinical practice.

## Neurochemistry of ADHD

### Overview

Current neurochemical models of ADHD are centered on putative dysfunctions in the dopaminergic and noradrenergic systems, which regulate attention, impulsivity, and executive control. Additionally, serotonin has been implicated in modulating mood and impulsive behavior, while glutamate and gamma-aminobutyric acid (GABA) participate in cortical excitability and the regulation of many neural networks. Drugs approved to treat ADHD (e.g., psychostimulants and atomoxetine) act by increasing synaptic catecholamine availability, improving prefrontal function, and current findings indicate that ADHD reflects a complex interaction among multiple neurochemical systems.

### Dopamine

The dopaminergic system plays essential roles in the regulation of attention [56,57], motor neuron control [58,59], spatial working memory [60], arousal [61–63], motivation, reward, and pleasure [64–68]—all domains relevant to ADHD pathophysiology [8,10]. Dopamine also participates in neurodevelopmental processes, guiding the maturation of fronto-striatal circuits relevant for attentional control [69–71]. Recent experimental evidence in rodents shows that dopamine neurons in the ventral tegmental area confer motivational value to actions, whereas those in the substantia nigra mediate action-specific learning [72].

Dopamine exerts its effects by binding to five receptor subtypes, grouped into D1-like (D1 and D5) and D2-like (D2, D3, and D4), which mediate distinct physiological responses. In humans, behavioral models and neuromodeling studies suggest an inverted-U relationship between dopaminergic signaling—particularly through D1 receptors in the prefrontal cortex—and cognitive performance. Intermediate D1 activation optimizes cognitive function, while both insufficient and excessive signaling impair it [73,74]. This dynamic helps explain why both stress/fatigue and dopamine hyper/hypoactivation can degrade performance in ADHD.

Several animal models reproduce dopaminergic components of the ADHD-like phenotype. In the spontaneously hypertensive rat (SHR) model—the most used animal model of ADHD [75–77]—neurochemical studies describe alterations in dopamine release, storage, and metabolism in the prefrontal cortex and striatum [78,79], paralleling observations reported in individuals with ADHD [80,81]. Complete deletion of the dopamine transporter (DAT) leads to a marked increase in extracellular dopamine and a hyperlocomotion phenotype [82], whereas partial DAT knockdown results in behavioral hyperactivity [83]. In contrast, DAT overexpression in transgenic mice disrupts dopaminergic regulation and alters responses to psychostimulants [84]. Together, these findings provide evidence that presynaptic dopaminergic alterations contribute to ADHD-like behaviors.

In the SHR model, behavioral and electrophysiological studies also reveal reduced phasic dopaminergic responses in the striatum, associated with less efficient stimulus-dependent release and atypical modulation by methylphenidate, although strain-dependent differences have been noted [85,86]. Another study reported no alterations in basal dopamine levels measured by *in vivo* microdialysis, suggesting that dysfunction is more evident in dynamic release mechanisms (evoked/phasic) than in tonic signaling. Factors such as age, brain region, and methodology may account for discrepancies [87]. Overall, dopaminergic dysfunction in animal models does not follow a single direction but emerges as a spectrum, combining hyper- or hypofunctional basal activity with reduced phasic signaling, ultimately producing less informative dopaminergic output and deficits in control and regulation characteristic of ADHD.

Experimental studies in humans with ADHD consistently demonstrate alterations in motivational and inhibitory control processes, both of which are modulated by dopamine. In delay discounting tasks, individuals with ADHD tend to favor smaller immediate rewards over larger delayed rewards, reflecting heightened impulsivity and a preference for immediate gratification [88,89]. This pattern, replicated across multiple cohorts, has been interpreted as a consequence of reduced dopaminergic signaling within reward circuits, which diminishes the salience of future reinforcers [68].

In the domain of inhibitory control, individuals with ADHD show poorer performance on stop-signal and Go/No-Go tasks, characterized by increased commission errors and greater variability in response times [90–92]. These results align with dopamine’s role in executive control and inhibitory processing. Yet, cognitive factors may modulate these effects: for instance, in pediatric samples, group differences in delay discounting disappear after adjusting for IQ, suggesting that intelligence can influence the expression of such deficits [93]. Moreover, some cohorts show no significant differences between ADHD and controls [94–96]. Thus, while behavioral evidence supports an important role for dopamine in motivation and inhibitory control in ADHD, inconsistencies highlight the need for more standardized experimental approaches.

Biochemical studies conducted in humans present heterogeneous findings. A meta-analysis found no significant differences in urinary dopamine levels or its metabolites [dihydroxyphenylalanine, dihydroxyphenylacetic acid, and homovanillic acid (HVA)] between individuals with ADHD and controls [97]. Pooled effect sizes were small and nonsignificant, with little to moderate heterogeneity across studies. Evidence from plasma dopamine levels was inconsistent, with one study reporting higher levels in ADHD [98] but not replicated [99]. Data from cerebrospinal fluid were limited to three studies of HVA, which showed mixed results [100–102], preventing firm conclusions. More recent metabolomic studies also found no significant associations with main dopamine-related metabolites [103–105]. However, some work has found reduced levels or altered metabolism of tyrosine, a precursor for dopamine synthesis [106,107], reinforcing the importance of further investigating metabolic pathways associated with dopaminergic neurotransmission.

Positron emission tomography (PET) studies in ADHD have yielded mixed findings. Some investigations have reported reduced D2/D3 receptor density and/or DAT availability within neural circuits involved in reward and motivation [68,108,109]. While reductions in DAT availability could theoretically increase extracellular dopamine due to reduced reuptake, the functional implications of these findings for ADHD are complex and remain debated [110]. However, other studies have not identified robust baseline differences in striatal DAT availability [111–113]. One study demonstrated a significant increase in DAT binding in the right caudate of adults with ADHD, confirming abnormal striatal DAT binding and supporting the notion that DAT dysregulation represents an important component of ADHD pathophysiology [114]. A longitudinal study also found DAT up-regulation after 12 months of methylphenidate in the caudate, putamen, and ventral striatum. Importantly, pretreatment differences between ADHD and controls were not significant, suggesting adaptive or compensatory mechanisms that complicate cross-sectional findings [115]. Overall, these findings indicate that while there is evidence of dopaminergic dysregulation in ADHD, PET results remain heterogeneous, likely influenced by factors such as study protocol, medication history, sample characteristics, and comorbidities.

One of the most clinically consistent indications of dopaminergic involvement in ADHD comes from pharmacology, as the primary medications approved for the disorder act on the dopaminergic system. Psychostimulants such as amphetamines and methylphenidate, as well as some antidepressants, increase synaptic dopamine availability and yield robust improvements in attention, impulsivity, and overall functioning, as consistently demonstrated in randomized clinical trials [116,117]. Methylphenidate, one of the most widely used treatments for ADHD, acts by blocking DAT-1 and inhibiting dopamine reuptake, thereby increasing synaptic availability of the neurotransmitter. This blockade is correlated with beneficial behavioral effects, supporting the dopaminergic hypothesis of ADHD [118,119].

Lisdexamfetamine dimesylate (LDX) through its active metabolite, d-amphetamine, exerts stimulant effects through DAT inhibition and activation of the trace amine-associated receptor 1, thereby regulating dopamine reuptake and promoting its release into the synaptic cleft [120]. Experimental studies in animal models further demonstrate that LDX produces a sustained increase in extracellular dopamine levels in the striatum and prefrontal cortex, corroborating its functional action on DAT [121,122].

Among antidepressants, bupropion is notable for acting as a non-selective inhibitor of monoamine transporters, including DAT, thus reducing dopamine reuptake [123,124]. Meta-analytic evidence suggests that bupropion may confer a modest benefit for ADHD symptoms in adults compared with placebo, although the quality and consistency of the evidence remain limited, and its efficacy appears inferior to that of stimulant medications [117,125].

Taken together, pharmacological evidence strongly supports the dopaminergic hypothesis of ADHD, showing that enhancing synaptic dopamine availability is consistently associated with improved clinical outcomes. Nonetheless, the heterogeneity of human and animal findings underscores that dopaminergic dysfunction is multifaceted and influenced by genetic factors. Future work must clarify how the dopamine system interacts with other neurotransmitters and adapts to long-term treatment, paving the way for more precise and personalized therapeutic strategies for ADHD.

### Noradrenaline

The noradrenergic system plays a central role in physiological regulatory networks. The locus coeruleus (LC), located in the dorsal pons of the brainstem, is the brain’s primary noradrenergic nucleus, with widespread projections to most regions of the central nervous system [126], including the cortex, hippocampus, and amygdala—key areas implicated in the pathophysiology of ADHD [127,128]. Norepinephrine released by the LC modulates several functions that are often impaired in ADHD, such as arousal and attention [129,130], memory [131–133], the sleep-wake cycle [134,135], and the stress response [136,137]. Its action also follows the inverted-U principle described above for dopamine [138–140].

Beyond these functional and behavioral effects, the noradrenergic system significantly influences synaptogenesis [141,142] and neurogenesis [143,144], supporting the notion that noradrenaline is critical for neurodevelopment. Catecholaminergic cell groups emerge early in human embryos [145], shaping the maturation of other brain regions [146]. Consequently, disruptions in the LC or noradrenergic signaling, particularly during critical developmental periods, may contribute to the causal pathways underlying neurodevelopmental disorders, including ADHD.

Some animal models of ADHD suggest dysfunctions in the LC-noradrenaline system. For example, SHRs exhibit alterations in noradrenaline signaling [147], supporting a noradrenergic basis for ADHD-like behaviors in this model [146]. Similarly, in the coloboma mouse, chemical lesioning of the LC-noradrenaline system with DSP-4 [*N*-(2-chloroethyl)-*N*-ethyl-2-bromobenzylamine hydrochloride] reduced locomotor hyperactivity, highlighting a contribution of noradrenergic signaling to ADHD-related symptoms [148]. Animals treated with monosodium glutamate (MSG), a compound that induces ADHD-like behavior, showed a significant reduction in noradrenaline compared with the control group [149–151], also pointing to noradrenergic abnormalities in ADHD.

The LC-noradrenaline system plays a central role in regulating pupillary responses [152]. In humans, pupillometry studies have shown that individuals with ADHD exhibit a significantly larger tonic pupil diameter and a reduced stimulus-evoked phasic pupil dilation compared with those without ADHD [153], as well as greater pupil dilation in response to happy—but not fearful, angry, or neutral—faces [154]. These pupillary diameter alterations in ADHD have been linked to impaired executive functions [155].

Neuroimaging findings are scarce. One volumetric magnetic resonance imaging (MRI) study reported a pontine reduction adjacent to the LC [156]. PET studies have reported mixed findings. Two studies found no significant differences in norepinephrine transporter (NET) availability in ADHD cases [157,158]. A third study demonstrated that patients with adult ADHD have decreased NET availability in relevant regions of interest for attention, which was more pronounced in the right hemisphere, compared with healthy controls. In exploratory analyses, lower NET availability in right fronto-parietal-thalamic-cerebellar regions was associated with more omission (but not commission) errors in a sustained attention task and greater electroencephalogram theta current density in the right dorsolateral prefrontal cortex [159].

In biochemical studies, a meta-analysis of 28 investigations comparing monoamines, their metabolites, and metabolic enzymes between individuals with and without ADHD identified alterations in the noradrenergic system [97]. Patients with ADHD had higher urinary noradrenaline levels than controls, whereas plasma noradrenaline levels showed no differences. For the main noradrenaline metabolite, normetadrenaline, baseline urinary differences lost significance after Bonferroni correction. Another well-studied metabolite, 3-methoxy-4-hydroxyphenylethylene glycol (MHPG), was significantly lower in the urine of ADHD patients, and platelet monoamine oxidase (MAO) activity was also reduced. Paradoxically, despite cross-sectional evidence associating low MHPG levels with ADHD, stimulant treatment trials have shown that symptom improvement correlates with further reductions in urinary MHPG excretion [97,160,161]. Collectively, the significant findings for noradrenaline, MAO, and MHPG suggest that reduced MAO activity may impair noradrenaline degradation, leading to lower MHPG levels, and that the combined ‘low MAO-high noradrenaline-low MHPG’ profile—given that a substantial fraction of urinary MHPG originates from central noradrenaline metabolism [162]—could be further investigated as a potential biochemical signature of ADHD [97].

Consistent indication of noradrenergic involvement derives from both mechanistic insights from preclinical research and pharmacological interventions targeting this system. Notably, the stimulation of postsynaptic α2A adrenergic receptors is thought to be an important mechanism for optimizing executive function [163,164]. Noradrenergic modulators such as atomoxetine, methylphenidate, amphetamines, guanfacine, and clonidine have shown beneficial effects in ADHD, although their mechanisms are complex and involve actions on multiple neurochemical systems [117,165]. Earlier pharmacological studies using tricyclic antidepressants (TCAs), including nortriptyline and desipramine, also reported improvements in core ADHD symptoms, providing additional support for the involvement of noradrenergic pathways [166,167]. However, TCAs also affect serotonergic signaling, underscoring that treatment effects in ADHD likely arise from overlapping neurochemical mechanisms.

Building on this, recent developments suggest that therapeutic effects in ADHD may benefit from the coordinated modulation of multiple monoaminergic systems rather than selective targeting of a single neurotransmitter. Centanafadine, a novel triple reuptake inhibitor acting on norepinephrine, dopamine, and serotonin transporters, has shown clinically meaningful reductions in ADHD symptoms in open-label and randomized controlled trials, with efficacy comparable to established treatments in some analyses [168–172]. Although still under investigation and not yet widely incorporated into clinical practice, such compounds highlight a potential shift in pharmacological strategies—from single-target approaches to broader monoaminergic engagement—particularly in light of the limited and inconsistent efficacy observed with more selective serotonergic agents (see below).

### Serotonin

The serotonergic system (5-HT) also has a central role in regulating many neurophysiological processes important to ADHD such as arousal [173], attention [174,175], impulsivity control [176,177], and emotional control [178–181]. These actions are mediated by multiple 5-HT receptor subtypes; each activates specific cellular signaling pathways that produce distinct functional effects in the tissues where it is expressed. Currently, seven receptor families (5-HT_1_-5-HT_7_), comprising 14 subtypes, are reported to be expressed in the brain [182,183], allowing serotonin to exert a diverse range of actions. Through these receptors, serotonergic neurotransmission may influence many regions of the brain, such as modulating neuronal excitability in cortical and subcortical areas [184], control of cortical network activity [185], and modulating pyramidal cell membrane potential in the hippocampus [186]. In the context of neurodevelopment, serotonin is critical for processes such as neuronal proliferation [187–189], migration [190–193], and synaptic formation [194].

Early disruptions in the serotonergic system have been suggested to alter brain circuit formation and the developmental trajectories of frontostriatal and corticolimbic systems [195,196]. The diverse effects of 5-HT in the brain are due to the diversity of presynaptic and postsynaptic 5-HT receptors in multiple brain regions, their expression on multiple cell types, their dimerization with other serotonergic receptors, and their diverse secondary messenger signaling [197,198]. Considering this, changes in this system can lead to a hyper- or hypoarousal state, affecting attention, impulsivity, and other behaviors.

Animal models reinforce the potential association of serotonin and ADHD. In homozygous serotonin transporter knockout rodents, inhibitory control was improved rather than impaired, indicating that altered serotonin transporter function can modulate impulse-control phenotype [199]. Pharmacological and genetic manipulations of 5-HT signaling during development further demonstrate its influence on attentional and impulsive behaviors. For instance, neonatal habenula lesions induce an imbalance between serotonin and dopamine, leading to hyperactivity [200], while altered serotonergic binding in the caudate-putamen and nucleus accumbens after dopaminergic lesions has been linked to motor hyperactivity [201]. Moreover, pharmacological interventions such as chronic L-deprenyl [202], acetyl-L-carnitine [203], serotonin-norepinephrine reuptake inhibitors [204], or prepuberal subchronic administration of methylphenidate and atomoxetine [205] can modulate impulsivity, locomotion, attention, and forebrain monoamine content in ADHD animal models, with methylphenidate additionally producing long-term behavioral effects while atomoxetine primarily affects neurochemical parameters. Environmental and behavioral manipulations also highlight serotonin's involvement. For instance, social deprivation induces long-term neurobehavioral alterations relevant to developmental psychiatric disorders [206,207], and the combination of caffeine consumption with physical exercise improves olfactory and recognition memory, increases hippocampal and prefrontal serotonin, and enhances synaptic plasticity in the SHR model [208].

In humans, evidence from clinical and neuroimaging studies shows that children and adolescents with both conduct disorder and ADHD and adults with ADHD exhibit differences in the availability of serotonergic transporters [209,210]. In a population-based neuroimaging study, higher attention problem scores were associated with smaller cortical surface area and greater cortical thickness across specific serotonin-related regions [211]. Acute tryptophan depletion (ATD), a method that transiently lowers central serotonin level since tryptophan is its essential amino acid precursor, has been shown to influence multiple behavioral and neural processes in individuals with ADHD. While ATD increased reactive aggression and aggressive decision-making in young people, its effects in adults appear to be more complex, with reduced reactive aggression that is inversely modulated by the baseline trait of impulsivity [179,212]. ATD also altered resting-state DMN connectivity, suggesting that serotonin contributes to large-scale brain network organization in ADHD [213].

Interestingly, serotonergic modulation appears to affect attention differently across development. For example, in children, diminished serotonin was linked to fewer lapses of attention [175], whereas in adults, ATD led to increased omissions and fewer correct responses during attentional tasks [174]. ATD did not significantly alter electrodermal activity, a measure of physiological arousal, suggesting that the effects of serotonin depletion may be more pronounced in behavioral and cognitive domains than in physiological arousal [180]. Finally, ATD did not alter verbal declarative memory in young individuals with ADHD, suggesting that serotonin’s contribution may be more critical for attentional and emotional regulation than for memory functions [214]. Taken together, these findings suggest that the impact of serotonergic modulation in ADHD may vary by age, domain, and behavioral predisposition (e.g., aggressivity) with stronger influences on attentional and emotional regulation than on memory or physiological arousal.

Although dopamine and norepinephrine remain the primary therapeutic targets in ADHD, interest in serotonin as a potential pharmacological pathway has increased. Serotonin agonists such as azapirones have been evaluated in randomized controlled trials. A systematic review reported mixed findings: most studies did not differ from methylphenidate, one favored the stimulant, and the available data were insufficient for a robust meta-analytic conclusion. The authors therefore considered the clinical benefit of these agents to be uncertain [215]. More recent systematic evaluations indicate that, despite convergent genetic, neurobiological, and preclinical evidence, pharmacological manipulation of serotonergic pathways has thus far yielded inconsistent efficacy results in ADHD patients [216,217]. Stimulants such as methylphenidate may indirectly influence serotonergic neurotransmission [218,219] and remain the most strongly supported treatments for reducing core symptoms [117,220]. Taken together, current evidence suggests serotonergic involvement in ADHD pathophysiology, yet no medications directly targeting this system have received regulatory approval.

### Glutamate

Glutamate is the main neurotransmitter acting predominantly at excitatory synapses [221]. It is essential for synaptic transmission [222,223], learning [224], and memory [225] and plays a key role in the regulation of mood [226] and sleep [227]. It signals through ionotropic receptors, specifically the α-amino-3-hydroxy-5-methyl-4-isoxazolepropionic acid receptors (AMPARs), *N*-methyl-d-aspartate receptors (NMDARs), kainate receptors, and orphan delta receptors, and through metabotropic glutamate receptors (mGluRs). NMDARs, in particular, are critical for synaptic plasticity and learning [224,228–230]. During development, glutamate supports synapse formation and stabilization, regulating postsynaptic density assembly and neural circuit remodeling [231].

Dysregulation of glutamate receptors during critical developmental periods can lead to neuropathologies and impair cognitive functions such as attention and behavior, which are relevant to ADHD [232,233]. One possible mechanism linking glutamate to ADHD is the interaction between glutamate transmission and the dopaminergic system. The combined evidence suggests that a hypodopaminergic state in ADHD may lead to increased activity of NMDARs/AMPARs, resulting in a subsequent increase in glutamate output [230]. Additionally, glutamate release in striatal regions facilitates dopamine release through NMDARs [234].

Several animal models of ADHD have demonstrated alterations in the glutamatergic system. In DAT knockout mice, hyperactivity can be exacerbated by NMDAR blockers and attenuated by drugs that facilitate glutamatergic transmission [235]. In SHRs, multiple dysfunctions have been described, including impaired NMDAR function in the prefrontal cortex [236], altered expression of glutamate receptor genes [237], and a hyperfunctional glutamatergic system characterized by increased release and aberrant uptake of glutamate [238]. Reduced expression of Grin1 and Gria1 in the nucleus accumbens has also been observed in SHR [239], while expression of Grin3a in the prefrontal cortex differs between SHR (a model of the combined subtype) and Wistar-Kyoto (WKY) (a proposed model of the inattentive subtype), suggesting that glutamatergic alterations may be linked to distinct ADHD symptom profiles [240]. Supporting the interaction between glutamate and dopamine signaling, SHR exhibits enhanced glutamate-induced dopamine release in the substantia nigra compared with WKY controls [241].

Beyond receptor-level changes, alterations in glutamate transport also contribute to ADHD-like phenotypes. Mice with reduced expression of the glial glutamate transporter (GLT1), expressing only ∼20% of normal levels, survive into adulthood and display core ADHD-like behaviors, including hyperactivity, impulsivity, and memory deficits [233]. In pharmacological models, rats exposed to MSG and displaying ADHD-like behavior showed elevated brain glutamate protein content compared with controls [149].

Other experimental manipulations confirm the role of glutamatergic dysregulation. In 6-OHDA-lesioned rats, a positive allosteric modulator of AMPARs reduced locomotor activity in a dose-dependent manner [242]. In double mutant mice lacking actin depolymerizing factor and n-cofilin, an ADHD-like phenotype characterized by hyperlocomotion, impulsivity, and working memory deficits was reversed by methylphenidate or blockade of glutamatergic transmission [243]. Finally, in DAT mutant rats, in addition to hyperactivity and cognitive and social impairments, the mGluR2/3 antagonist LY341495 normalized hyperactivity without affecting extracellular dopamine levels [244]. Taken together, these findings suggest that dysregulation of the glutamatergic system—including receptor dysfunction, transporter deficiency, altered gene expression, and disrupted glutamate-dopamine interactions—may play an important role in the emergence of ADHD-like phenotypes across animal models.

In humans, a meta-analysis of 33 magnetic resonance spectroscopy (MRS) studies—including 874 patients with ADHD and 775 control participants—highlighted the relevance of glutamate-glutamine imbalance in ADHD [245]. Primary analyses revealed that children with ADHD had higher concentrations of a composite measure of glutamate and glutamine (Glx) in the right medial frontal area. More recently, a single-site study reported that adults with ADHD exhibited lower glutamate levels in the posterior cingulate cortex (PCC) compared with controls [246]. These lower concentrations suggest that a glutamatergic imbalance in PCC may contribute to the pathogenesis of ADHD or represent a correlate of its persistence into adulthood.

In biochemical studies, children with ADHD showed significantly lower serum glutamine and higher glutamate levels compared with controls, resulting in a reduced glutamine-to-glutamate ratio. These alterations were associated with ADHD symptom severity and may reflect dysregulated glutamatergic neurotransmission [247]. Supporting this finding, another study in children and adolescents also reported elevated serum glutamate levels in individuals with ADHD [248]. In contrast, a study including children with ADHD and controls found no significant difference in serum glutamate levels between groups [249]. An imbalance in biomarkers related to glutamate receptor signaling was identified in the plasma of children with ADHD, characterized by low levels of cyclin-dependent kinase 5 and high levels of microtubule-associated protein 2, suggesting implications for the disorder's etiopathogenesis [250].

There is growing evidence that major pharmacological treatments for ADHD, including psychostimulants (methylphenidate, amphetamines) and atomoxetine, also modulate the glutamatergic system, particularly via NMDARs. In preclinical studies, methylphenidate enhanced NMDAR-mediated neuronal excitability in the prefrontal cortex, suggesting an indirect influence on glutamatergic neurotransmission in addition to catecholaminergic effects [251]. Furthermore, atomoxetine exhibits NMDAR antagonist activity at clinical concentrations, which may contribute to its therapeutic effects [252].

Extending these findings, a study in adolescent rats demonstrated that chronic atomoxetine administration reduced NMDAR 2B subunit protein levels in the striatum and hippocampus, as well as NET levels in the hippocampus. Importantly, these effects persisted two months after treatment discontinuation, with reduced expression of NMDAR subunit genes in the mesencephalon and striatum and decreased NMDAR protein levels in the striatum and hippocampus. Atomoxetine also altered the levels of key synaptic proteins, including synaptophysin and synaptosomal-associated protein of 25 kDa, suggesting long-lasting, region-specific reductions in glutamatergic transmission beyond acute NET inhibition [253].

In MRS studies conducted in children and adolescents with ADHD, the effects of methylphenidate on glutamate levels were heterogeneous. Some studies reported significant reductions in glutamate-to-creatine ratio (Glu/Cr) in specific regions, including the fronto-cerebellar circuit [254], the left and right prefrontal cortex [255], and the bilateral amygdala [256]. Additionally, one study found a significant increase in Glx/Cr in the white matter posterior to the left dorsolateral PFC [257]. In contrast, other studies in children did not detect significant changes in glutamate levels in the right PFC, left striatum, occipital lobe, anterior cingulate cortex (ACC), or bilateral globus pallidus after methylphenidate treatment [258,259].

In adults with ADHD, MRS studies consistently reported no significant changes in glutamate or related metabolites following methylphenidate use. One study observed reduced glutamate levels in the left striatum compared with controls, but this effect was attributed to ADHD itself rather than methylphenidate treatment [260]. After an acute methylphenidate challenge, no differences in Glx were found in the medial prefrontal cortex between medicated and unmedicated individuals [261]. Similarly, the only double-blind, placebo-controlled study in adults found no differences in Glx in the ACC or cerebellum after 12 weeks of treatment [262].

Finally, therapeutic alternatives targeting the glutamatergic system directly have been explored as adjunctive or alternative treatments for ADHD. A positive allosteric modulator of the AMPAR (Org-26576) demonstrated preliminary efficacy in a translational preclinical and clinical study, further supporting the glutamatergic pathway as a therapeutic target in ADHD [242]. In randomized clinical trials, non-competitive NMDAR antagonists, such as amantadine and memantine, demonstrated improvements in ADHD symptoms [263–265].

### GABA

GABA represents the predominant inhibitory neurotransmitter in the central nervous system. However, during early development, it can exert excitatory actions on immature neurons, promoting primitive patterns of neural activity that support neuronal growth and synapse formation [266]. In addition to regulating the excitation-inhibition balance in cortical and subcortical circuits [267–269]—primarily through interneurons that fine-tune neural network activity—the GABAergic system participates in critical networks related to attention [270], regulation of the sleep-wake cycle [271,272], and stress responses [273–275], mediated by the action of ionotropic (GABA_A) and metabotropic (GABA_B) receptors. Although findings remain mixed [276–278], recent evidence points to GABA as a relevant neurotransmitter in the pathophysiology of ADHD, with studies indicating alterations in its concentration and function across different brain regions [278,279].

In animal models of ADHD, such as the SHR, reduced extracellular levels of GABA in the hippocampus have been demonstrated, associated with relevant behavioral alterations, including hyperactivity and learning/attention deficits [280]. These findings suggest that GABAergic deficits may causally contribute to ADHD-like behavioral manifestations. However, other experiments using the SHR model and receptor-binding assays did not observe differences in GABA binding measures in regions such as the prosencephalon, cerebellum, and pons-medulla when compared with controls [281].

The role of GABA in the medial PFC (mPFC) has also been explored. GABAergic blockade through acute bicuculline infusions induced inattentiveness in the delayed non-matching-to-sample task, as well as impairments in sociability in the social preference test [282]. Recent evidence employing chemogenetics showed that selective activation of parvalbumin interneurons in the ACC reduced impulsivity [283]. Finally, the effect of GABA on attentional deficits appears to be bidirectional within the mPFC, since both muscimol-induced hypoactivation (a GABA-A agonist) and picrotoxin-induced disinhibition (a GABA-A antagonist) of this region impaired attentional performance [283,284].

The literature provides heterogeneous neuroimaging evidence regarding GABA alterations in ADHD as measured by MRS. Pediatric studies targeting the sensorimotor cortex or striatum typically report reduced GABA concentrations [278,279,285,286], whereas investigations focusing on the ACC have generally yielded null results [286]. Beyond resting-state measurements, task-based approaches have shown that individuals with ADHD display blunted increases in GABA during attention control tasks compared with controls [287]. Moreover, long-term effects of stimulant treatment appear to modulate inhibitory neurochemistry, as adults who initiated methylphenidate use in early adolescence exhibited reduced basal prefrontal GABA+ compared with those who began later or remained stimulant-naïve [261]. The variability across studies likely reflects methodological differences, including voxel placement, field strength, and whether GABA is reported as GABA+ or as a ratio to creatine (GABA/Cr), as well as other technical considerations [288].

Despite the possible relation between GABA and ADHD symptomatology, high-quality syntheses and guidelines support stimulants first and certain non-stimulants second, with GABAergic medications not being usually recommended for core ADHD symptoms [116,289,290]. Benzodiazepines, as well as other GABAergic agents, lack evidence of benefit for ADHD and may carry risks including sedation, cognitive impairment, and paradoxical reactions in children, limiting their role in this context [116,289–292]. Overall, although no GABAergic pharmacological targets have demonstrated consistent efficacy on the core symptoms of ADHD, converging neurobiological evidence, including neurochemical, neuroimaging, and genetic findings, implicates GABA in the disorder's pathophysiology.

## Structural and functional neuroimaging of ADHD

### Structural findings

Early volumetric MRI studies revealed that children with ADHD exhibited globally reduced total brain volume relative to typically developing controls (−3.2% cerebral volume and −3.5% cerebellar volume) [293,294]. Subsequent analyses consolidated these observations, demonstrating bilateral volume reductions in the nucleus accumbens, amygdala, caudate, hippocampus, and putamen, with the largest effect in the amygdala (Cohen’s *d* = −0.19), consistent with potential alterations in motivation, reward system, emotional dysregulation, and learning [127]. Other studies reported that these subcortical volumetric differences in children were accompanied by cortical morphological alterations, particularly in frontal, cingulate, and temporal regions, with the largest significant effect observed for total surface area (Cohen’s *d* = −0.21) [128]. Nonetheless, these effects seem to be small to moderate. The largest population-based study to date, the ABCD cohort, replicated only modest differences (Cohen’s *d* = −0.11 to −0.06) after rigorous adjustment for confounders, underscoring the small magnitude of the differences between cases and controls [295].

Regarding neurodevelopment, longitudinal evidence has indicated that ADHD is associated with a delay in the maturation of cortical architecture. The attainment of peak cortical thickness may occur approximately 3 years later in individuals with ADHD, most prominently in prefrontal association regions supporting executive control [296]. Cross-sectional analyses from the ENIGMA-ADHD consortium further reinforce the delayed neurodevelopment hypothesis, demonstrating that volumetric reductions in cortical and subcortical regions are greatest in childhood and attenuate in adolescence and adulthood [127,128]. For example, ENIGMA-ADHD analyses showed reduced cortical thickness in the fusiform gyrus and temporal pole in children with ADHD, but not in adults [128]. Collectively, these findings suggest that the ADHD brain phenotype changes across development, with structural differences most pronounced in childhood and attenuating with age.

In adults, different case–control studies using voxel-based and surface-based morphometry have indicated modest reductions in PFC, ACC, and parietal cortex, as well as variations in basal ganglia volumes [297–302]. Nonetheless, large-scale analyses do not demonstrate convergent cortical or subcortical effects in adults with ADHD [127,128,303]. Given the limited sensitivity of these volumetric measures in this group, neuroimaging modalities more sensitive to white matter structure become particularly informative. Diffusion tensor imaging (DTI) studies have detected widespread white matter alterations in adults with ADHD, including differences of fractional anisotropy in the corpus callosum, left uncinate fasciculus, inferior fronto-occipital fasciculus, and superior longitudinal fasciculus. These microstructural abnormalities are correlated with persistent symptoms and impaired cognitive control [304–306]. Notably, many DTI studies are rated as having lower methodological quality, and in high-quality study subsets, some effects were no longer significant, suggesting that methodological factors may influence the results [305]. Overall, the findings suggest that while gross morphological differences diminish with age, widespread disruptions in white matter connectivity may represent more stable neurobiological markers in adults with ADHD.

### Functional findings

Some of the earliest functional MRI (fMRI) investigations of ADHD sought to characterize the mechanisms of inhibitory control, frequently employing the Go/No-Go paradigm, which yielded variable findings [307–315]. In adolescents, initial work identified reduced activation in prefrontal regions, supporting early descriptions of ADHD as a disorder marked by ‘hypofrontality’ [308]. Interestingly, later event-related studies in adolescents revealed greater activation in prefrontal regions during inhibition tasks [316] and atypical recruitment of diffuse frontotemporal regions under inhibitory demands [307,309]. Although no unified result emerged from these early works [317], these foundational investigations provided the basis for later models of brain network dysfunctions in ADHD.

One prominent hypothesis posits that the DMN plays a central role in ADHD symptomatology [318–321]. The DMN is an intrinsically organized set of brain regions that are more synchronized at rest and are involved in self-referential thinking and internally oriented cognition, with the PCC and precuneus serving as main integrative hubs [322]. An abnormal interplay between the DMN and task-positive cognitive control networks (e.g., dorsal attention network, ventral attention network, salience network) may lead to insufficient suppression of DMN activity during goal-directed tasks requiring executive control, an anti-correlation that some studies found to be decreased in individuals with ADHD [323–332]. In addition to this inter-network view, ADHD patients may exhibit altered connectivity within the DMN, reduced in the core hub (PCC) and increased within other subsystems (temporal pole-inferior frontal gyrus) [333].

Another main area of fMRI studies on ADHD concerns the reward system. Task-based fMRI studies investigating reward mechanisms consistently implicate mesolimbic circuits in ADHD, with reports of attenuated ventral striatal responses during reward anticipation on Monetary Incentive Delay paradigms in adolescents and adults. In these studies, lower striatal activation was related to greater hyperactive/impulsive symptoms [334,335]. These patterns suggest that reward anticipation in ADHD is characterized by attenuated anticipatory signaling, reflecting reduced motivational salience of forthcoming rewards, whereas the reward itself (outcome) can be relatively preserved or even heightened, including increased striatal and orbitofrontal responses [336–338]. However, results are heterogeneous, with some studies revealing preserved anticipatory responses but altered outcome-related signaling [336,337].

Complementing these findings, a high-resolution fMRI study imaged orbitofrontal responses to small versus large expected rewards in adolescents. While all participants showed stronger responses to large rewards, adolescents with ADHD displayed exaggerated signaling relative to controls, which correlated with hyperactive/impulsive symptoms and normalized with higher IQ [338]. These effects appear sensitive to biological and contextual influences: striatal responsivity varies by *DAT1* genotype, while stimulant pharmacotherapy, particularly methylphenidate, has been shown to enhance or normalize activity within reward and attention-motivation networks during incentivized tasks [336,339,340].

In addition to disrupted network interactions, convergent functional region-specific alterations have emerged as potential hallmarks of ADHD, with some fMRI meta-analyses reporting reduced activation in areas such as the inferior frontal cortex, supplementary motor area, insula, and basal ganglia [341–344]. Notably, an fMRI meta-analysis of pharmacological effects demonstrated that the use of methylphenidate enhanced the activity in two of these regions, the inferior frontal cortex and the insula, suggesting that stimulant medication may at least partially normalize the neural deficits observed in ADHD [345]. More recent analyses suggest that these effects, particularly within the salience network (including the insula) and sensorimotor regions, may reflect a modulation of arousal and motivational processes rather than a direct restoration of the specific functional deficits described above [346].

Nonetheless, other quantitative syntheses have failed to show spatially convergent abnormalities in ADHD across resting-state fMRI and task domains, underscoring substantial heterogeneity in samples and methods [317,321,347]. Overall, although current evidence suggests functional abnormalities in regions such as the inferior frontal cortex and basal ganglia, as well as in DMN-control network and reward system dynamics, findings across fMRI studies remain heterogeneous, and future research should prioritize harmonized analytic approaches and larger samples to resolve inconsistencies.

## Genomics of ADHD

Converging evidence indicates that the etiology of ADHD is neurodevelopmental, highly polygenic, and multifactorial [8,10,41,53,54,348]. In recent years, intense research has focused on unraveling its molecular basis through genomics [10,349]. This endeavor aims to achieve several goals, including identifying genes that could reveal biological pathways with potential for novel therapeutic targets and developing polygenic score-based algorithms to predict clinical outcomes (e.g., symptom severity, persistence, etc.) and treatment responses.

ADHD is one of the neuropsychiatric conditions with the highest heritability estimate [349], a measure referring to the proportion of variation in a given phenotype within a population that is attributable to genetic heritable factors [350]. Twin studies are a traditional method for assessing heritability by comparing the concordance of a trait between identical (monozygotic) and non-identical (dizygotic) twin pairs [351]. For ADHD, twin studies conducted across various European countries estimate a weighted average heritability of 76%, a value consistent across children, adolescents, and adults [349,352].

**Figure 3:**
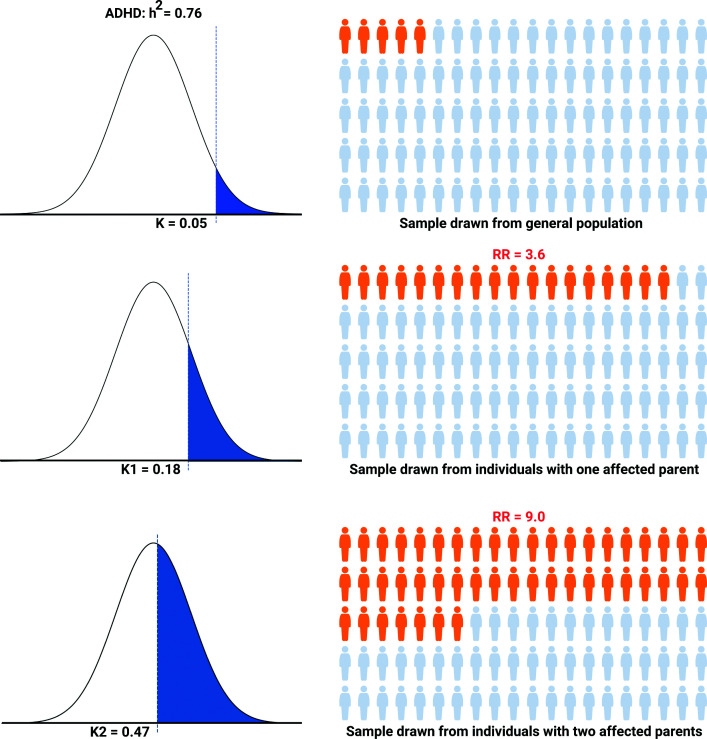
Risk, risk in relatives, and the liability threshold model for ADHD In a population where 5 in 100 individuals have ADHD, the population-wide prevalence independent of age is *K* = 0.05. Assuming the liability to disease follows a normal distribution, the top 5% of the distribution (shown in green) is bisected by the liability threshold, representing individuals with ADHD. Now, consider the lifetime risk of ADHD in individuals with one affected parent (K1). In this scenario, 18 in 100 individuals are affected. Under the same diagnostic criteria, the liability distribution shifts, expanding the blue area to include the top 18% of the distribution. Next, consider the lifetime risk of ADHD in individuals with two affected parents (K2). Here, 47 in 100 individuals will have the condition. Again, applying the same diagnostic criteria as in the general population, the liability distribution shifts further, with the blue area now covering the top 47% of the distribution. The risk ratio (RR) values for individuals with one affected parent (RR1 = 3.6) and for those with two affected parents (RR2 = 9.0) were calculated to be consistent with a heritability estimate (h^2^) of 0.76. These estimates were generated using the CHARRGe Shiny application available at shiny.cnsgenomics.com/CHARRGe. Adapted from [353].

This high heritability underscores the significant role of genetic factors, which aggregate within families. For example, the prevalence of ADHD is expected to be almost 18% among individuals with a first-degree relative who has the disorder, and this prevalence might increase to over 47% when two first-degree relatives are affected (Figure 3) [353]. These estimates are supported by data from a large population-based sample, which showed that the hazard ratio for ADHD—compared with the frequency of ADHD in relatives of unaffected individuals—is 70 for monozygotic twins, around 8 for dizygotic twins and full siblings, between 2 and 3 for half-siblings, 2 for first cousins, and 1.5 for second cousins [354].

Due to its high polygenic burden, there is considerable interest in identifying genetic variations associated with ADHD using genome-wide association studies (GWAS). GWAS analyses millions of genetic variants across many individuals to identify associations with a trait of interest, whether it is a molecular trait or endophenotype (e.g., gene expression), physiological variable, disease, or condition [355]. GWAS signals are starting points rather than endpoints, requiring integrative functional analyses to uncover the mechanisms they implicate. By charting unbiased associations across the genome, GWAS highlights biological pathways and regulatory architectures that might otherwise escape attention, thereby accelerating the translation from statistical signal to physiological insight [350]. This hypothesis-free approach has greatly advanced our understanding of the neurobiology of neuropsychiatric disorders [54,55,356–360].

As sequencing technologies become increasingly accessible for large-scale studies, whole-exome sequencing (WES) and whole-genome sequencing represent natural extensions of GWAS. These approaches enable the detection of rare and structural variation that array-based designs may miss, thereby improving the resolution of genetic discovery in ADHD.

### Mapped genes and pathways

In the context of ADHD, three GWAS meta-analyses are particularly notable [53,54,361]. The first study included 12 cohorts, comprising 20,183 individuals with ADHD and 35,191 controls [53]. This meta-analysis was the first to identify genetic variants that surpassed the genome-wide significance threshold of *P* < 5 × 10^−8^. The identified variants were in 12 independent *loci*, with associations enriched in evolutionarily constrained genomic regions, loss-of-function-intolerant genes, and brain-expressed regulatory networks. Among the genes identified, a region spanning *ST3GAL3* (ST3 Beta-Galactoside Alpha-2,3-sialyltransferase 3) and *KDM4A* (lysine demethylase 4A) stood out. These genes are strongly associated with educational attainment and depression—phenotypes that are correlated with ADHD [362,363].

Another important finding was *FOXP2* (Forkhead Box P2). This gene is associated with language and writing skills and has also been linked to psychiatric, personality, and neuroimaging traits [364]. In a large Integrative Psychiatric Research Consortium (iPSYCH) GWAS evaluating genetic differences among childhood (*n* = 14,878), persistent (*n* = 1473), and late-diagnosed (*n* = 6961) ADHD cases, alongside 38,303 controls, *FOXP2* variants were associated with late-onset ADHD [42].

As a continuation, the second GWAS meta-analysis expanded the sample size to 38,691 individuals with ADHD and 186,843 individuals without the disorder, revealing 27 significant loci [54]—more than twice the number reported in the previous study. Positional annotation and gene-based analysis highlighted 76 plausible causal risk genes, including *PTPRF* (protein tyrosine phosphatase receptor type F), *SORCS3* (sortilin related VPS10 domain containing receptor 3), and *DCC* (DCC netrin 1 receptor), which encode integral components of the postsynaptic density membrane, and *FOXP1*, a novel ADHD locus. FOXP1 and FOXP2, both transcription factors that can heterodimerize to regulate gene expression in the brain, have been implicated in neurodevelopmental disorders through highly penetrant and equivalent missense variants [365]. Notably, *MEF2C* (myocyte enhancer factor 2C) was also among the identified genes and has been linked to brain white matter microstructure and various neuropsychiatric disorders in the context of ADHD [366].

The set of 76 genes associated with ADHD in the latest GWAS meta-analysis [54] is enriched for genes up-regulated during early embryonic brain development and shows strong enrichment for genes identified in multiple GWAS of cognition-related traits [363,369,370] and reproductive phenotypes [371]. Of these, nine genes play roles in synaptic function, either as integral components of the synapse or as participants in its organization and processes, including chemical synaptic transmission (e.g., *PTPRF*, *SORCS3*, and *DCC*).

**Figure 4:**
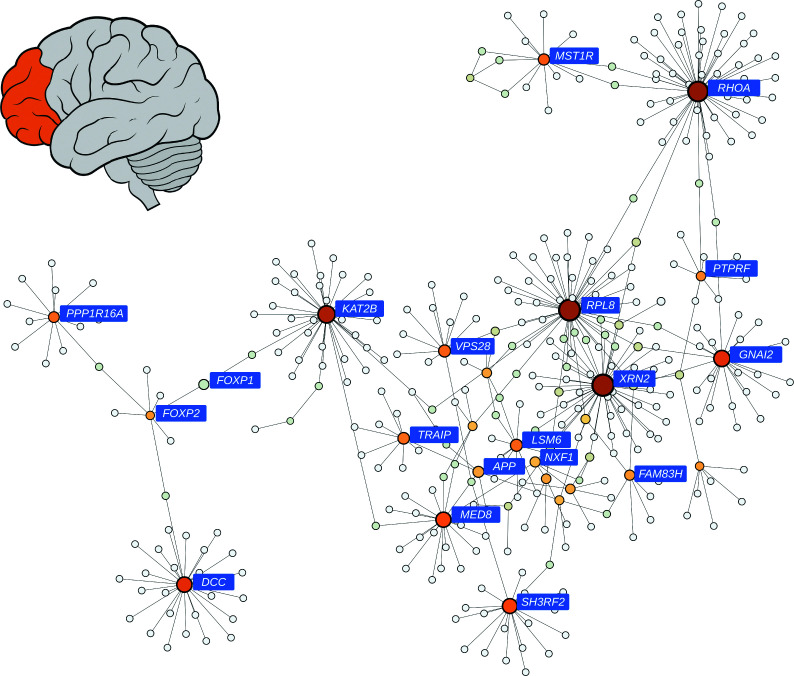
Tissue-specific protein-protein interaction (PPI) network derived from ADHD core genes filtered for expression in the frontal cortex Large orange nodes denote the most highly connected (hub) proteins; smaller light orange/gray nodes are their first interactors. Edges represent reported or predicted physical interactions between proteins. Node size was scaled to interaction degree, and labels indicate selected genes with high centrality (e.g., RHOA, RPL8, XRN2, KAT2B, and DCC) or context importance (e.g., FOXP1). This network highlights modules and hub proteins that may coordinate molecular processes relevant to ADHD in the frontal cortex. The Network was assembled using DifferentialNet [367] as an interactions database and NetworkAnalyst [368] as visual software.

The study employed multiple approaches to investigate tissue and cell type specificity. These analyses revealed that ADHD-associated genes are enriched among those highly expressed in the brain, particularly in the cortex. Heritability analyses further supported this finding, showing enrichment of variants in genes specifically expressed in the frontal cortex (Figure 4). Cell type-specific analyses also indicated that ADHD-associated variants are enriched in genes expressed in key neuronal populations, including both excitatory and inhibitory neurons, with a notable association with genes expressed in dopaminergic neurons of the midbrain [54].

The most recent GWAS meta-analysis evaluated whether integrating dimensional symptom measures with case–control designs can enhance gene discovery in ADHD [361]. In a large dataset comprising 70,953 individuals and 290,134 cross-rater assessments of childhood symptoms, no genome-wide significant loci emerged when symptoms were analyzed in isolation. When these data were combined with the prior GWAS of clinical ADHD diagnosis [54], however, the effective sample size increased substantially, yielding 39 independent loci, 17 of which had not surpassed genome-wide significance in the diagnostic analysis alone. Importantly, the genetic correlation between symptoms and diagnosis was indistinguishable from unity, reinforcing the view that ADHD reflects the extreme of a continuous liability distribution. Gene-prioritization approaches further highlighted biologically plausible candidates, including genes not previously implicated in ADHD, such as *EMCN* (endomucin) and *TCF12* (transcription factor 12), with enrichment for frontal cortical expression and synaptic pathways. Together, these results illustrate that dimensional information, even when individually noisy, can materially improve discovery by increasing statistical power and sharpening the link between polygenic burden and neurobiological mechanisms.

WES studies have also provided important evidence for the polygenic nature of ADHD. Both the iPSYCH single-site GWAS [42] and the latest ADHD GWAS meta-analysis [54] reported an increased burden of rare protein-truncating variants in ADHD, with the latter also identifying a set of risk genes enriched for causal common variants (8895 ADHD cases and 9001 controls). Notably, the *SORCS3* gene was implicated by both common and rare variant analyses [54]. Another study conducted WES in 152 families—each comprising a child with ADHD and both biological parents—and found a significant enrichment of rare and ultra-rare *de novo* gene-damaging mutations in ADHD cases compared with unaffected controls [372]. When these results were combined with an independent case–control WES cohort (3206 ADHD cases and 5002 controls), *KDM5B* (lysine demethylase 5B) emerged as a high-confidence ADHD risk gene. *KDM5B* encodes a histone-modifying enzyme, and genetic variation in this gene has been associated with autism [373], developmental disorders [374], and cognitive function [375]. Using a Bayesian framework, the authors estimated that approximately 1057 genes (95% CI 219–2791) contribute to ADHD risk [372]. This high predicted polygenicity aligns with the latest ADHD GWAS meta-analysis, which estimated that about 7300 (standard deviation = 324) common variants influence the disorder [54].

More recently, a large exome-sequencing effort analyzing 8895 ADHD cases against an expanded control set exceeding 50,000 individuals identified exome-wide significant associations in *MAP1A* (microtubule associated protein 1A), *ANO8* (Anoctamin 8), and *ANK2* (Ankyrin-2), with odds ratios ranging roughly from five to over fifteen [376]. The study also showed that rare deleterious variants were more frequent among individuals with co-occurring intellectual disability, whereas ADHD without intellectual disability still displayed an excess burden relative to controls. Importantly, although variants of this magnitude can approach penetrance levels compatible with quasi-monogenic presentations at the individual level, they are observed in only a small fraction of cases. At the population scale, their contribution therefore remains limited, and risk continues to be distributed across a very large number of genes.

### Shared genetics with other traits

GWAS have significantly enhanced our understanding of the relationships between ADHD and various phenotypes. Many of these associations are well recognized in clinical practice, such as the high prevalence of mood disorders among individuals with ADHD and their struggles with academic performance [3]. Consistently, ADHD exhibits significant positive genetic correlations with conditions such as depression, bipolar disorder, autism, schizophrenia, substance use disorders, insomnia, obesity, suicide behaviors, and chronic pain [53–55,363,377,378]. In contrast, it shows negative genetic correlations with educational attainment, household income, intelligence, and other cognitive traits [53,54,379,380].

Approaches aimed at uncovering shared genetic factors are also helping to clarify the continuity of ADHD across the lifespan. A GWAS meta-analysis investigating the genetic underpinnings of ADHD throughout life stages conducted both separate and combined analyses of childhood and persistent ADHD, including 17,149 cases and 32,411 controls [41]. The study found a strong shared genetic basis between childhood and adult persistent ADHD, with a genetic correlation close to 0.8. Similar results were observed in an independent study comparing individuals with childhood ADHD (*n* = 14,878), persistent ADHD (*n* = 1473), and late-diagnosed ADHD (*n* = 6961) against 38,303 controls. The genetic correlation was ∼0.8 between childhood and persistent ADHD, ∼0.8 between persistent and late-diagnosed ADHD, and ∼0.7 between childhood and late-diagnosed ADHD [42]. Together, these findings reinforce the view of persistent adult ADHD as a neurodevelopmental condition [4] and extend the concept of a shared genetic architecture to a lifespan framework.

Genetic correlation analyses across life stages also revealed distinct patterns of overlap with other traits—childhood ADHD showed greater overlap with hyperactivity and autism, whereas late-diagnosed ADHD was more strongly correlated with depression [42]—aligning with the clinical profiles typically observed in children/adolescents and adults [48–50]. Moreover, strong concordance with GWAS of quantitative population measures of ADHD symptoms supports the view that a clinical diagnosis reflects the extreme expression of continuous heritable traits such as inattention, hyperactivity, and impulsivity [53].

Contextualizing these associations within a multivariate framework, a recent large-scale genomic modeling across 14 psychiatric disorders identified five latent dimensions: compulsive, psychotic, neurodevelopmental, internalizing, and substance use [381]. Within this latent structure, ADHD anchors the neurodevelopmental factor with an exceptionally high loading (λ = 0.91), defining the domain alongside autism and Tourette’s. Strikingly, its architecture reveals a unique pleiotropic profile: unlike other neurodevelopmental conditions, ADHD exhibits a significant secondary loading on the substance use factor (λ = 0.34) and shares substantial genetic liability with internalizing disorders. Notably, the genetic correlation with PTSD (*r*_g_ = 0.74) and major depression (*r*_g_ = 0.60), for example, substantially exceeds that observed with autism (*r*_g_ = 0.43), indicating that the biological vulnerability for ADHD is not narrowly confined but rather constitutes a pervasive genomic liability for behavioral dysregulation that transcends traditional diagnostic boundaries.

GWAS not only quantifies overall biological correlations driven by shared genetic components but, through local genetic correlation analyses, can pinpoint specific genomic regions that link traits [350,382]. For example, one study found that ADHD is associated with inheriting fewer alleles related to high intelligence, whereas autism presents a more complex profile, combining both high- and low-intelligence alleles along with additional autism-specific risk variants unrelated to intelligence [383]. This is especially noteworthy because, while ADHD and autism are positively correlated [53,54], ADHD shows a negative genetic correlation with intelligence, whereas autism shows a positive one [369].

This locus-specific heterogeneity is consistently observed across different biological domains. For instance, a recent LAVA-based study examining ADHD and cortisol variability found no significant global genetic correlation yet identified multiple genomic regions with significant local correlations, including loci with opposite directions of effect [384]. These findings illustrate how biologically meaningful relationships may be obscured at the genome-wide level when aggregating across heterogeneous loci, reinforcing the importance of regional analyses to uncover underlying mechanisms.

Beyond this regional heterogeneity, local genetic analyses have also begun to reveal biologically coherent pathways underlying shared genetic architectures. In the context of suicidality, loci jointly associated with ADHD and suicidal ideation mapped to genes with enriched expression in brain regions such as the hypothalamus and frontal cortex and were implicated in processes related to neurodevelopment, ion transport, and transcriptional regulation [385]. These associations showed both concordant and discordant directions of effect across loci, and several remained significant even after conditioning on major confounders such as depression and post-traumatic stress disorder, indicating partially independent shared genetic mechanisms.

A recent study [377] further highlighted the strong genetic and biological links between ADHD and chronic pain. Twelve genomic regions made substantial contributions to the overall genetic correlations. Shared neurodevelopmental mechanisms likely drive this relationship, as key regions and genes associated with both conditions are enriched in neurodevelopmental pathways, including those involved in neuron projection morphogenesis and nervous system development. These findings suggest that chronic pain may partly originate from neurodevelopmental processes and underscore its clinical relevance in patients with ADHD, particularly regarding the potential influence of ADHD medications on pain outcomes. Although larger, stratified datasets and functional studies are needed to validate and expand upon these findings, they pave the way for exploring novel pharmacological strategies and a deeper understanding of the shared biological mechanisms connecting ADHD and pain.

### Causal inference

Beyond quantifying genetic correlations, GWAS enable the assessment of whether observed relationships arise from vertical pleiotropy (i.e., due to causal effects) or horizontal pleiotropy, in which shared genetic influences without direct causality play a role [350]. For instance, Mendelian randomization (MR) studies have provided evidence supporting a causal relationship between ADHD liability and an increased likelihood of tobacco smoking initiation, smoking heaviness, and cannabis initiation as well as a decreased likelihood of tobacco smoking cessation. Conversely, the genetic liability to substance use does not appear to cause ADHD, suggesting that associations in this direction are likely attributable to horizontal pleiotropy [386].

Moreover, the most comprehensive MR study to date expanded these findings by evaluating causal relationships between ADHD liability and 124 traits spanning anthropometry measures, cognitive function, early-life exposures, education attainment, college completion, employment, lifestyle and environment, longevity, and psychiatric and mental health [387]. The present study provided evidence for a causal effect of ADHD genetic liability on decreasing average total household income and increasing the lifetime number of sexual partners. It also identified positive effects of anthropometric traits, such as predicted arm mass and weight, and time spent watching television on ADHD liability. Furthermore, the results revealed bidirectional negative effects between ADHD and educational outcomes, including years of schooling and the age of completing full-time education. Similar bidirectional effects were observed between ADHD liability and age at first sexual intercourse, as well as past tobacco smoking in non-heavy smokers. These findings underscore the complex interplay between genetic and environmental factors—many of which are themselves genetically influenced—that contribute to ADHD and its broader impact on life outcomes.

## Other ‘omics’

Several studies have investigated ADHD using other ‘omics’ approaches in both human samples and animal models. While these studies are typically smaller in scale than GWAS, they still provide insights into environmental and molecular mechanisms contributing to the disorder. They encompass the epigenome, reflecting environmentally driven modifications; the transcriptome and proteome, which capture tissue- and cell-specific molecular signatures; and the metagenome, which examines the influence of the gut microbiome.

Epigenomic research in ADHD has primarily focused on 5-methylcytosine levels, assessed either at birth or during childhood, often in longitudinal cohorts. Early studies identified specific genes with differential methylation patterns, though replication has been inconsistent, likely due to methodological differences [388–391]. Nevertheless, two specific gene findings are worth mentioning: *VIPR2* (vasoactive intestinal polypeptide receptor 2) replicated between studies [389,391] and associated with brain functioning and *ST3GAL3*, previously linked to intellectual disability [392] and associated with ADHD through GWAS [54] and animal models [393].

Large meta-analyses have shown enrichment for genes related to brain development and function at birth but not in later childhood [394]. In adults, findings instead implicated immune- and neuron-related genes, as well as loci linked to autoimmune disease, cancer, and neuroticism [395,396]. More recent work highlights the dynamic nature of the methylome, showing that epigenome-wide association studies (EWAS) findings at one developmental stage may not generalize to others [397]. Global methylation levels (GMe) were also explored in ADHD patients, being lower than in controls, as well as in women [398]. In addition, a polygenic risk score (PRS) for ADHD was negatively correlated with GMe, showing the intersection between ADHD genomics and the epigenome [398]. Taken together, these studies underscore the need for caution when interpreting epigenomic signals, particularly given sex-specific effects and developmental timing.

Transcriptomic research has aimed to clarify the functional roles of ADHD risk genes identified by GWAS. Early studies of peripheral blood mononuclear cells (PBMCs) in persistent ADHD implicated the ubiquitin-proteasome system [399], while later analyses reported differentially expressed genes overlapping with other psychiatric and comorbid conditions [400]. MicroRNA studies have revealed disrupted myo-inositol signaling [401] and immune-related pathways [402]. More recently, a comprehensive multi-step study in PBMCs identified seven modules of co-expressed genes enriched for genetic and epigenetic risk signatures, implicating pathways related to gene regulation, epigenetics, and immune function [403]. These findings highlighted candidate genes and pathways, underscoring the potential of blood-based expression studies and the value of multi-omics integration for advancing our understanding of ADHD biology.

Transcriptome-wide association studies (TWAS) integrating expression quantitative trait loci with GWAS signals [53] demonstrated enrichment for genes expressed in fetal astrocytes, neurons, and microglia/macrophages [404]. Other TWAS following the same base GWAS further mapped gene expression to brain and blood tissues, implicating pathways such as glycan degradation, viral myocarditis, and endocytosis [405], and identified brain-region-specific candidate genes with potential causal roles that overlap with psychiatric and cognitive traits [406]. A follow-up TWAS leveraging the gene-based ADHD GWAS showed enrichment for genes expressed in the brain, particularly in the cortex [54].

Proteomic studies in ADHD remain scarce. Animal models include WKY rats, SHR rats, Sprague-Dawley (SD) rats, and thyroid hormone-responsive protein-overexpressing (THRSP-OE) mice. Striatal protein profiling in WKY, SHR, and SD rats found no significant changes following maternal separation [406]. In contrast, THRSP-OE mice showed hippocampal Wnt signaling dysregulation linked to impaired attention, memory deficits, and altered neural stem cell activity [407], as well as striatal Snap25 overexpression, suggesting SNARE (Soluble *N*-ethylmaleimide-sensitive factor Attachment protein REceptor) complex dysfunction and neurotransmitter dysregulation [408]. Only one study has investigated the impact of ADHD treatment on the proteome, showing that methylphenidate altered cortical protein expression in WKY rats, with enriched effects on components of synaptic transmission (e.g., SNARE) that were also observed in clinical samples [409,410].

Microbiome and metagenome studies support a role for gut microbiota in ADHD pathophysiology via the gut–brain axis. Microbiota assessed via 16s measurements have shown differences between ADHD cases and typical development controls in diversity levels [411–413] and specific taxa [411–417]. However, meta-analysis results only support differences in the Shannon index of alpha diversity and the increase in the *Blautia* genus in ADHD patients [418].

On the other hand, case–control studies exploring shotgun metagenomics report reduced abundance of beneficial taxa such as *Faecalibacterium* and *Veillonellaceae* and increased levels of O*doribacter* and *Enterococcus*, with functional pathway analysis implicating neurotransmitter metabolism, including serotonin and dopamine [419]. Larger cohorts confirmed significant differences in microbial diversity and identified potential microbial biomarkers of ADHD spanning nine representative species that best explain the differences [420]. Integrative metagenomics-metabolomics approaches further revealed disruptions in nicotinamide metabolism, while fecal microbiota transplantation from ADHD patients into rats induced hyperactivity-like behaviors, suggesting a potential causal contribution of the microbiome in ADHD-related traits [421]. Nonetheless, the causal direction between microbiome variability and ADHD symptoms remains unclear. It is also important to note that the studies cited here have several limitations, including small sample sizes and substantial heterogeneity in diversity metrics and taxonomic classification, which increase the risk of false-positive associations [422]. Overall, this is an evolving field that would benefit from more standardized guidelines and evaluation criteria.

## Environmental contributions

Environmental factors play a key role in ADHD, particularly through non-shared influences that uniquely affect individuals, as demonstrated in twin studies [352,423]. While psychosocial adversities—such as poverty and negative parenting—may not directly cause ADHD, they can shape its course and prognosis, especially in Western contexts where most studies have been conducted [8,424]. Early developmental periods are especially sensitive to environmental factors, including low birth weight, prematurity, maternal stress, and prenatal exposure to substances such as tobacco and alcohol [425–429].

Environmental pollution has also emerged as a factor of growing interest in neurodevelopmental research [430–432]. Recent meta-analyses report associations between exposure to lead and traffic-related pollutants, particularly fine particulate matter (PM2.5) and nitrogen dioxide (NO_2_), and increased likelihood of ADHD diagnosis or greater symptom burden, although effect sizes are typically modest and findings vary across studies depending on exposure window, assessment strategy, and control for socioeconomic and contextual factors [430,431,433]. Importantly, the current body of evidence does not support the view that environmental pollution acts as a deterministic cause of ADHD [430,431]. Rather, it is more consistent with a multifactorial liability model in which environmental exposures may contribute to risk magnitude or symptom expression, particularly among individuals with pre-existing neurobiological or genetic vulnerability.

How these exposures become biologically embedded is therefore a central question. Some of these environmental effects may be mediated by epigenetic mechanisms, such as DNA hypo- or hypermethylation, which influence gene expression and have been linked to ADHD status and symptom variability [395,397,398,434]. Additionally, somatic mutations occurring during early embryonic development may lead to brain mosaicism and help explain why monozygotic twins can differ phenotypically [435,436]. These stochastic biological processes suggest that part of the variance typically attributed to ‘environmental’ factors may, in fact, originate biologically [437,438].

An additional dimension involves gene-environment correlation (rGE), whereby genetic factors shape the environments individuals are exposed to. This can occur passively (e.g., disorganized households reflecting inherited traits), evocatively (e.g., impulsive children eliciting negative parental responses), or actively (e.g., selecting peer groups or environments aligned with genetically influenced tendencies) [439–442]. Recognizing the role of rGE is essential not only for informing clinical strategies, such as psychoeducation and family-based interventions, but also for interpreting associations between ADHD and environmental exposures correctly.

These interpretative challenges are evident in studies of environmental risk factors potentially involved in ADHD. Paracetamol use during pregnancy is a relevant example, as it is one of the few environmental exposures classified as having Class I evidence for ADHD risk [428]. A study investigating ADHD and pain-related traits found that, although the strong genetic correlation between ADHD and chronic pain argues against a purely causal environmental effect, follow-up gene-drug enrichment analyses indicated that paracetamol may also modulate the expression of neurodevelopmentally relevant genes [377]. This suggests that genetic predisposition and xenobiotic influences are not mutually exclusive. Instead, they may operate in parallel, with both complex genetic effects and direct environmental mechanisms contributing to risk. While this interpretation is consistent with preclinical evidence showing that paracetamol can disrupt neurodevelopmental processes [443], it also helps explain why epidemiological studies have yielded mixed results regarding the association between prenatal paracetamol exposure and ADHD [444,445]. Recent syntheses reach differing conclusions [446–448], largely reflecting recurring methodological challenges, including uncertainty in exposure measurement (over-the-counter use, dose, duration, and timing), confounding by indication (e.g., pain, fever, and infection), and shared familial, genetic, and environmental factors.

Applying a different evaluative strategy, a 2025 assessment using the Navigation Guide framework [446] concluded that the overall body of evidence is consistent with an association between prenatal paracetamol exposure and increased probability of neurodevelopmental disorders, despite substantial heterogeneity across study designs and definitions. In contrast, a meta-analysis published the same year [447], incorporating explicit bias analyses, estimated a small increase in ADHD risk (odds ratio ≈1.17) and did not find clear evidence for autism, emphasizing the potential impact of residual confounding and exposure misclassification. Furthermore, a recent umbrella review [448] rated confidence in many existing reviews as low or critically low, noted extensive overlap among primary studies, and concluded that the available evidence does not establish a clear link between prenatal paracetamol exposure and ADHD or autism. Importantly, associations observed in whole-cohort analyses frequently did not persist in sibling-comparison designs, supporting a relevant contribution of shared familial, genetic, or environmental influences.

This interpretation is reinforced by a large national population study [444] including approximately 2.5 million children, which detected modest associations in conventional models but found no significant association in analyses comparing differentially exposed siblings. Such designs substantially reduce confounding from familial liability and are widely interpreted as indicating that signals detected in traditional cohort analyses may not reflect direct causal effects of the medication. Importantly, even if interpreted causally, effect sizes of this magnitude imply a small absolute difference. Assuming a baseline ADHD prevalence of ∼5%, an odds ratio of 1.17 would translate into an estimated risk of approximately 5.9% among exposed offspring—roughly one additional case per 100 pregnancies. Thus, the vast majority of exposed children would not develop ADHD, underscoring the importance of contextualizing relative risks within clinical and public-health perspectives.

Taken together, the literature calls for caution. If a causal effect exists, it is likely small and highly sensitive to confounding and exposure uncertainty. Part of this confounding may itself reflect rGE, whereby familial liability associated with ADHD risk can also influence patterns of medication use during pregnancy. Clinical decisions are therefore framed within risk-benefit reasoning, balancing hypothetical medication-related risks against the known consequences of untreated maternal conditions.

Distinct from rGE, gene-environment interactions (G×E) occur when genetic factors modify how individuals respond to environmental exposures—a concept highly relevant to ADHD [449]. Classical epidemiological models suggest that genetic and environmental influences may act independently or synergistically. However, such models often assume independence between genes and environment, a condition rarely met in practice, as many exposures—such as trauma or socioeconomic status—are themselves influenced by genetics [363,450]. As mentioned above, even prenatal paracetamol exposure may reflect shared genetic liability, given overlaps between ADHD and chronic pain [377].

The polygenic architecture of ADHD, involving thousands of common variants of small effect [53], poses challenges for detecting robust G×E effects. Candidate gene studies have yielded few replicable results [451], but genome-wide interaction studies hold promise, although none have yet been published for ADHD. An increasingly adopted strategy is the polygenic risk score by environment (PRS×E) framework, and a growing body of work has begun to test these interactions in the context of ADHD.

A study of over 33,000 individuals found that ADHD-PRS and psychosocial risk factors independently contributed to ADHD risk, with no significant interaction [452]. Similarly, a Danish population study showed only limited interaction between ADHD-PRS and psychosocial adversities or birth-related risks [453]. In U.S. preadolescents, ADHD-PRS predicted suicidal ideation independently of early life stress [454], while in adolescent cohorts, additive—but not interactive—effects of PRS and family conflict were observed [455]. Several studies instead support rGE over GxE interactions. For instance, genetically influenced ADHD traits predicted lower parental involvement and inconsistent discipline [456], and individuals with higher ADHD genetic liability were more likely to experience maltreatment during childhood [457]. This bidirectional relationship—where maltreatment also increased ADHD risk—was further supported in a Brazilian birth cohort. In the present study, higher ADHD symptoms mediated the link between genetic risk and adversity exposure [442].

Nonetheless, evidence for genuine G×E interaction is emerging. In another Brazilian sample, ADHD-PRS significantly interacted with environmental stress, amplifying ADHD risk without evidence of rGE or similar effects for depression and anxiety [458]. Another study in Norway found that the influence of ADHD traits and PRS on academic performance varied across schools, with higher-performing environments buffering genetic risk [459]. Finally, spatial analyses in the United Kingdom revealed that the association between PRS and ADHD traits varies geographically, suggesting regional environments may shape genetic expression [460]. Consistent with this, recent work has shown that rGEs can extend beyond the family level to broader geographic and socioeconomic structures, influencing GWAS signals and heritability estimates [461]. Collectively, these findings highlight the multifaceted nature of gene-environment dynamics in ADHD, with evidence for additive effects, rGEs, and G×E interactions depending on the population, context, and outcome studied. Future research must continue integrating genetic and environmental data across developmental, social, and geographic domains to clarify ADHD etiology and improve clinical prediction.

## Translational and clinical implications

### From biology to ADHD risk prediction

Despite extensive research into the biological underpinnings of ADHD—spanning neurochemistry, neuroimaging, genomics, other omics, and more recently their integration—validated biomarkers for clinical use are still lacking [3,10]. This reflects the complexity of ADHD, a highly polygenic and multifactorial condition in which individual risk factors exert only very small effects. Consequently, there is no single ‘ADHD gene,’ in contrast with autism, where approximately 30% of cases can be traced to monogenic or oligogenic variants [462]. Given this landscape, a promising strategy for identifying clinically useful biomarkers in ADHD is the development of composite biological scores. For instance, no single neuroimaging finding has shown an effect size large enough to reliably distinguish individuals with ADHD from those without it. However, neuroimaging-based scores, or brain scores, could combine multiple small effects into a single metric. Large meta-analyses of neuroimaging studies provide the association coefficients that can be aggregated into such scores, allowing individuals to be placed along a risk distribution [463].

A recent study showed that an ADHD brain score for cortical surface area was higher in individuals with ADHD compared with controls [464]. However, this score was not associated with the number of ADHD symptoms or with clinical trajectories over time. While this approach is promising, current brain scores account for only a small fraction of the phenotypic variance [463]. Their performance relies heavily on the quality and statistical power of the meta-analyses used to derive effect sizes, which remain underpowered to capture the full spectrum of associations relevant to ADHD. In this sense, their current picture represents a tool that is relevant to study the relationship between neuroimaging features of a given phenotype (ADHD) and other traits.

An important innovation comes from circuit-based scores. For example, in depression and anxiety, functional brain circuit scores have been shown to identify clinically distinct biotypes that differ in symptoms, cognition, and treatment response [465]. This raises the possibility that future approaches in ADHD could leverage brain circuit scores as well as the integration of structural and functional features to enhance the predictive performance of composite biological scores.

Similarly, PRSs aim to capture the cumulative effect of numerous genetic variants of very small effects associated with a trait or disorder. They are derived from large GWAS—or from EWAS in the case of methylation risk scores—by summing the number of risk alleles carried by an individual, each weighted by its estimated effect size [350]. No single genetic variant has an effect size large enough to reliably distinguish individuals with ADHD, but PRSs provide a way to combine these small effects into a single quantitative measure.

**Figure 5:**
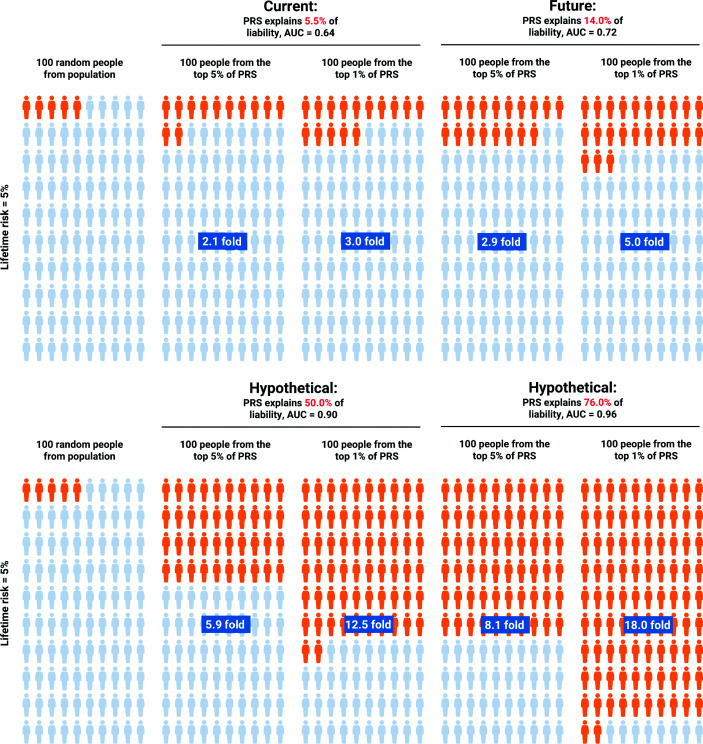
ADHD risk prediction using polygenic risk scores (PRS) under varying predictive scenarios Assuming a 5% lifetime prevalence of ADHD and current PGS explaining approximately 5.5% of variance in disease liability, the rates of ADHD cases (orange) are shown among 100 individuals selected at random from the population or from the top 5% or top 1% of PGS (top left). In this scenario, the area under the curve (AUC = 0.64) indicates a 64% probability that an individual with ADHD will have a higher PGS than an unaffected one. A future scenario assumes PGS explains 14% of liability, approximating current ADHD SNP-heritability, raising AUC to 0.72 (top right). Hypothetical scenarios illustrate upper bounds in predictive performance if PGS explained 50% (bottom left) or 76% (bottom right) of liability (AUC = 0.90 and 0.96, respectively), demonstrating that as PGS accuracy increases, individuals in the highest percentiles become progressively more enriched for ADHD risk. These simulations highlight the potential of increasingly powerful PGS to enable meaningful population stratification in ADHD. Adapted from [467,468].

In ADHD, PRSs have been associated not only with case–control status across independent samples but also with clinically relevant traits such as comorbidities, cognitive performance, executive function, and attentional measures, reflecting the disorder’s clinical profile [466]. As with brain scores, however, current PRSs explain only a modest proportion of phenotypic variance—about 5.5% in ADHD—limiting their predictive power [53]. Even under a highly hypothetical scenario where a genetic score captured the entire heritability of ADHD, only the extreme end of the distribution would be clinically decisive: for example, in the top 1% of the PRS distribution, ADHD would be expected in 92% of individuals (Figure 5). In fact, the strongest genetic information currently available still comes from family history [353]. As shown in Figure 3, individuals with two first-degree relatives with ADHD have almost a 50% risk of developing the disorder. Therefore, PRSs, like brain scores, are not suitable as stand-alone diagnostic tools but may ultimately contribute as one component of a multimodal framework, complementing family history (e.g., parental diagnosis) and standardized clinical assessments and serving as an additional pillar in the diagnostic process.

### Diversity as a source of innovation

Although GWAS (and other omics studies) have been pivotal in understanding complex behavioral conditions such as ADHD, they suffer from a strong Eurocentric bias [469–474]. Over the past five years, 85% of the samples included in major psychiatric GWAS meta-analyses were from individuals of European ancestry residing in high-income countries. For ADHD specifically, this percentage is almost 100%, underscoring a major gap in representativeness [473]. This perspective builds on recent work highlighting ancestral diversity as a critical determinant of gene discovery, biological interpretation, and the equitable translation of ADHD genomics [475].

Genomic diversity in ancestral and admixed populations is increasingly recognized as a source of underexplored genetic variation that can uncover biological mechanisms that would remain invisible in more homogeneous datasets [476]. A notable example is the discovery of loss-of-function mutations in *PCSK9* (proprotein convertase subtilisin/kexin type 9) among African Americans, which were associated with lower cholesterol levels and reduced cardiovascular risk [477]. These mutations had a combined frequency of 2% in African Americans but were rare in European Americans (<0.1%). This discovery led to the development of *PCSK9* inhibitors, such as alirocumab and evolocumab [478,479]. Similar patterns have emerged in other loci identified through the study of diverse populations. For instance, risk variants in *APOL1*, largely restricted to individuals of African ancestry, have revealed a trade-off between protection against trypanosomal infection [480] and increased susceptibility to kidney disease [481,482], reshaping our understanding of renal pathophysiology and informing ongoing therapeutic development [483]. Although these examples are not specific to psychiatry, they illustrate how diversity can generate findings with major translational impact—something needed in psychiatry and ADHD research.

Beyond limiting mechanistic discovery, Eurocentric bias in psychiatric genomics also contributes to the amplification of health disparities [473]. A clear example is the reduced portability of PRSs, whose predictive performance declines substantially as the genetic distance between the discovery and target populations increases [350,484]. This reduction in accuracy arises from differences in allele frequencies, linkage disequilibrium patterns, and environmental contexts across populations, leading to systematically poorer risk prediction in individuals of underrepresented ancestries. As a result, these groups, including individuals with ADHD, are less likely to benefit from advances driven by GWAS, such as risk stratification, early detection, and the development of precision medicine approaches. Expanding ancestral diversity in genomic studies is therefore essential from both ethical and public health perspectives, particularly in countries characterized by high levels of admixture and environmental heterogeneity [473,475].

Increasing diversity in genomic studies in psychiatry is a challenging but essential endeavor that demands substantial investments in human resources, equipment, and supplies. Moreover, long-standing analytical obstacles must be addressed. Historically, GWAS pipelines for data quality control and analysis often excluded individuals of non-European ancestry to form more homogeneous groups, thereby reducing the risk of false positives caused by population stratification [485–487]. Population stratification arises when systematic differences in allele frequencies exist between subgroups within a population due to ancestry [488]. For example, an allele with a higher frequency in individuals of African descent and a lower frequency in Europeans might appear to be associated with a trait if there is an imbalance in genetic ancestry between cases and controls. In such situations, genome-wide analyses of millions of genetic variants can yield hundreds of significant findings driven by ancestry differences rather than genuine biological effects linked to the trait under investigation [475]. This is particularly concerning for highly polygenic phenotypes like ADHD, where subtle effects spread across the genome can be easily confounded by ancestry-related artifacts if they are not handled properly.

Analytical advancements, such as mixed-model-based tests, now account for population structure, enabling the analysis of admixed individuals [489]. A groundbreaking study in psychiatry recently applied this approach, conducting the largest meta-analysis of major depression to date, which included over 5 million individuals, more than 1 million of whom were of diverse ancestry [357]. This trans-ancestry GWAS identified 697 variants and 308 genes associated with depression, implicating postsynaptic density, neuronal dysregulation, and amygdala involvement. Notably, half of these loci are novel, with 100 identified solely due to the inclusion of diverse samples. The findings are enriched for potential antidepressant targets and highlight opportunities for drug repurposing. Similarly, a multi-ancestry GWAS of bipolar disorder, including 158,036 cases and 2.8 million controls, identified 298 genome-wide significant loci, with evidence that some signals were ancestry-specific and that multi-ancestry data improved fine-mapping resolution and polygenic prediction [490]. Together, these studies show that incorporating ancestral diversity is not merely a matter of representation but a strategy that can expand locus discovery, refine biological inference, and improve translational potential. These examples highlight how diversity-aware analytic frameworks can generate discoveries that would probably remain undetected in exclusively European datasets, reinforcing their relevance for future ADHD genomics.

Further innovations, such as advances in local ancestry inference (LAI) methods, are transforming the inclusion of admixed samples in GWAS. Local ancestry refers to the ancestral origin of specific genome regions in an admixed individual. LAI methods estimate the ancestry of each haplotype—a physical grouping of genetic variants within the genome—by comparing individual genetic data with reference population panels derived from dense DNA sequencing data [491].

These algorithms have become integral to some GWAS pipelines. Admixture mapping, for example, serves as a powerful approach to identifying genetic variants associated with diseases or traits that exhibit ancestry-specific risk patterns [492,493]. Recent tools like the *Tractor* pipeline further enhance the ability to model admixture in genomic studies. *Tractor* systematically analyzes admixed populations and has shown promising performance in both simulated and real datasets [494]. This advancement enables researchers to conduct GWAS on ancestral tracts within a single cohort, reflecting the population from which the study sample was recruited. By generating ancestry-specific effect size estimates, *Tractor* enhances the detection of genetic associations, especially when gene effects vary across ancestries.

In ADHD, the potential of these methods remains vastly underutilized. While recent GWAS meta-analyses have begun to unravel the biological underpinnings of the disorder, nearly all of them rely on data from European populations. Diversity is the missing ingredient with the potential to unlock unprecedented advancements in the field and make precision psychiatry a reality worldwide. However, ADHD genomics still lags behind. To date, only one study has applied new approaches, such as Tractor, to ADHD research. The present study analyzed a sample of just over 500 Colombian and Mexican individuals [495]. Although it marked a significant milestone—as the first GWAS of admixed individuals with ADHD—its small sample size limited statistical power, precluding the identification of genome-wide significant variants. Expanding such efforts is essential to ensure that ADHD research reflects the global population it aims to serve. Only by incorporating diversity in genomics will we be able to fully uncover the clinical and biological complexity of ADHD, improve diagnostic tools across populations, and develop equitable, targeted interventions.

## Conclusions

### Methodological considerations and strength of evidence

Over the past decades, the biological investigation of ADHD has progressed from small, conceptually pioneering studies to large-scale collaborative science. This transition has profoundly reshaped how evidence is weighed, interpreted, and integrated across levels of analysis. Findings from early neuroimaging and experimental investigations remain essential for their historical and hypothesis-generating value. However, contemporary inference regarding robustness, reproducibility, and generalizability increasingly depends on meta-analytic efforts and international consortia, including initiatives such as ENIGMA-ADHD, which provide the statistical power required to detect the modest effects expected for a highly heterogeneous and polygenic condition.

The same evolution characterizes psychiatric genetics. Initial efforts centered on candidate-gene hypotheses inspired by prevailing neurobiological models. As data accumulated, it became evident that ADHD liability is far more polygenic and widely distributed across the genome than originally assumed. This realization drove the adoption of hypothesis-free genome-wide approaches and underscored the need for global collaborative frameworks capable of achieving the sample sizes required for reliable discovery, as exemplified by the pioneering work of large international consortia.

Yet, despite rapid progress in gene discovery, clinical heterogeneity remains one of the field's most formidable challenges. ADHD encompasses diverse developmental trajectories, symptom profiles, and patterns of comorbidity. Disentangling what is specific to ADHD from what reflects broader, shared psychopathological mechanisms is only now becoming feasible and will depend critically on coordinated investments in deep phenotyping, longitudinal designs, and harmonized integration of biological, clinical, and environmental data.

Importantly, effect sizes observed in psychiatric case–control contrasts are typically small. Differences in the range of *d* ≈ 0.2–0.3, such as those discussed in the neuroimaging subsection, imply substantial overlap between individuals with and without ADHD, emphasizing that these results illuminate population-level mechanisms rather than serving as diagnostic signatures. Recognizing this statistical reality is fundamental for a balanced and mature interpretation of biological findings.

### Future directions

Progress in ADHD biology will depend less on isolated discoveries and more on coordinated advances in scale, design, and integration across levels of analysis (Table 1). The polygenic architecture of the disorder requires increasingly large and collaborative efforts to refine risk loci, improve fine-mapping resolution, and connect association signals to biological function. At the same time, overcoming the historical Eurocentric composition of samples is essential both for scientific validity and for ensuring that emerging tools are broadly applicable across populations [475].

**Table 1:** Priorities for the next generation of biological research in ADHD

Domain	Current main limitations	Recommendations for future studies
Animal models	Most models reproduce restricted behavioral dimensions rather than the full clinical phenotype, variable construct validity, divergent underlying mechanisms, inconsistent pharmacological responses, and potential strain-related confounders [75–77,498].	Treat models as platforms to interrogate specific dimensions rather than as replicas of the disorder; align tasks with homologous human cognitive constructs; incorporate human genetic liability into experimental strategies; promote multi-laboratory replication; and develop cross-species and developmental frameworks.
Neuroimaging	Robust group-level differences but extensive overlap at the individual level; small effect sizes; diagnostic heterogeneity; medication and environmental confounding; predominance of cross-sectional contrasts; limited clinical translation [499].	Increase scale through harmonized consortia; prioritize longitudinal within-subject trajectories; invest in deep phenotyping; integrate imaging with behavioral and computational models; embed biomarkers in treatment studies; adopt preregistration, transparent pipelines, and external validation.
Genetics/Genomics	Individual loci account for small fractions of risk, persistent Eurocentric sampling, reduced portability of polygenic estimates, and difficulty moving from association to specific mechanisms (non-shared mechanisms) [350,473,475,500].	Increase recruitment in underrepresented and admixed populations; implement ancestry-aware designs and local ancestry inference; leverage diverse LD patterns for fine-mapping; integrate common and rare variation with functional annotation; strengthen equitable global collaborations.
Epigenomics/Transcriptomics	Strong tissue and developmental specificity; modest sample sizes; limited replication; challenges in separating cause from consequence [501].	Establish longitudinal cohorts beginning early in life; harmonize laboratory and computational pipelines; jointly model genetic liability and environmental exposures; prioritize replication across tissues and populations.
Microbiome/metagenomics	High heterogeneity in sampling and analytics; small cohorts; inconsistent microbial signatures; major influence of diet, medication, and shared environment; typically small variance explained; frequent causal overinterpretation [422].	Move toward large, standardized, and collaborative frameworks; incorporate family-based and longitudinal designs; predefine hypotheses and report effect sizes; integrate host genomics and metabolomics; apply formal causal inference strategies before translational claims.

Beyond genomic discovery, the field faces a shared challenge across experimental systems. Effect sizes are typically small, heterogeneity is substantial, and findings that are robust at the group level often show limited utility for individual prediction. Addressing these constraints will require harmonization of methods, deeper phenotyping, longitudinal designs, and explicit modeling of environmental and treatment-related influences.

Importantly, progress will also depend on strengthening bridges between domains. Genetic discoveries must increasingly inform experimental models; neuroimaging findings need integration with computational and behavioral frameworks; and emerging omics layers such as epigenomics and the microbiome require larger, standardized, and replicable infrastructures before causal or translational claims can be sustained.

Furthermore, artificial intelligence may accelerate this transition by enabling multimodal integration at a scale previously unattainable. Rather than functioning as an independent solution, AI frameworks will likely be most informative when embedded within well-characterized cohorts, transparent analytical pipelines, and clinically meaningful questions. In this context, predictive modeling may help identify probabilistic risk profiles, stratify patients, and refine hypotheses about treatment response, contributing to more precise and biologically informed psychiatry.

Finally, after more than 50 years in which the concept of endophenotypes has remained largely a ‘future perspective’ in psychiatry—since Gottesman first proposed it in 1973 [496]—we now face a more concrete possibility of identifying them and applying them to diagnosis and treatment. Recent conceptual advances have reframed endophenotypes as genetically influenced phenotypes linked not only to disease liability but also to treatment-related characteristics and their associated biological events [497]. The increasing resolution of omics technologies, combined with advances in cellular and molecular neuroscience, now enables a more precise characterization of biological pathways and mechanisms relevant to neurodevelopment. This evolving framework strengthens the search for ADHD endophenotypes and may help move the field beyond theoretical aspirations toward biologically informed markers capable of linking genetic liability to clinical expression and treatment response.

## Abbreviations

ACC: anterior cingulate cortex
ADHD: attention-deficit/hyperactivity disorder
ATD: acute tryptophan depletion
AMPARs: α-amino-3-hydroxy-5-methyl-4-isoxazolepropionic acid receptors
DAT: dopamine transporter
DMN: default mode network
DTI: diffusion tensor imaging
EWAS: epigenome-wide association studies
fMRI: functional MRI
FOXP2: Forkhead Box P
GABA: gamma-aminobutyric acid
GMe: global methylation levels
Glx: glutamate and glutamine
GWAS: genome-wide association studies
HVA: homovanillic acid
IQ: intelligence quotient
LAI: local ancestry inference
LC: locus coeruleus
LDX: lisdexamfetamine dimesylate
MBD: minimal brain dysfunction
MAO: monoamine oxidase
MAP1A: microtubule associated protein 1A
mPFC: medial PFC
MHPG: 3-methoxy-4-hydroxyphenylethylene glycol
MR: Mendelian randomization
MRI: magnetic resonance imaging
MRS: magnetic resonance spectroscopy
mGluR: metabotropic glutamate receptors
MSG: monosodium glutamate
NMDARs: *N*-methyl-D-aspartate receptors
NET: norepinephrine transporter
PBMCs: peripheral blood mononuclear cells
PCC: posterior cingulate cortex
PCSK9: proprotein convertase subtilisin/kexin type 9
PET: positron emission tomography
PRS: polygenic risk score
PTPRF: protein tyrosine phosphatase receptor type F
rGE: gene-environment correlation
SNARE: Soluble N-ethylmaleimide-sensitive factor Attachment protein REceptor
SORCS3: sortilin related VPS10 domain containing receptor 3
SHR: spontaneously hypertensive rat
SD: Sprague-Dawley
ST3GAL3: ST3 Beta-Galactoside Alpha-2,3-sialyltransferase 3
TCF12: transcription factor 12
TCAs: tricyclic antidepressants
TWAS: transcriptome-wide association studies
VIPR2: vasoactive intestinal polypeptide receptor 2
WES: whole-exome sequencing
WKY: Wistar-Kyoto
